# Bacterial chromosome conformation and cell-free gene expression in synthetic 2D compartments

**DOI:** 10.1038/s41467-025-65249-2

**Published:** 2025-11-14

**Authors:** Ferdinand Greiss, Shirley S. Daube, Vincent Noireaux, Roy Bar-Ziv

**Affiliations:** 1 Department of Chemical and Biological Physics, Weizmann Institute of Science, Rehovot, 7610001 Israel; 2 School of Physics and Astronomy, University of Minnesota, Minneapolis, MN 55455 USA

**Keywords:** Single-molecule biophysics, Nanobiotechnology, Biomaterials, Lab-on-a-chip, Chromosomes

## Abstract

The *E. coli* genome is encoded on a contiguous ~4.6 Mb-long DNA molecule, compacted inside a micron-cubed cell. When reconstituted in vitro, chromosomes expand in a bulk that is challenging for probing single-chromosome DNA transactions and conformational changes. Here, we report transplanting *E. coli* chromosomes into 2D semi-open microfluidic compartments, enabling exchange of conditions, stretching by electric field, mapping DNA-bound proteins, and cell-free transcription-translation at steady-state. We find transplanted chromosomes emerge as intact, compacted, blob-like structures, decorated with native proteins from the donor cell. The blobs include clusters of condensin proteins and exclude sparse bright ribosome foci, whereas RNA polymerases uniformly decorate the chromosome. Introducing a transcription-translation system, we measure genome-average transcription rates and image the birth of individual proteins from a reporter gene on the chromosome. Our data suggest a dilute regime without translational amplification or multiple synthesis events per gene. The removal of native proteins reveals a conformation transition from expanded to compacted state upon increased molecular crowding. Interestingly, transcription has a swelling effect, pushing the compaction transition to higher crowding levels. Our work opens a window into genome-scale DNA transactions outside a cell and helps tackle the bottom-up assembly of autonomous artificial cells.

## Abstract (web summary)

Chromosomes are long DNA polymers, compacted in cells and fragile once released from the host. Here, authors report on a method to gently transplant bacterial chromosomes into 2D microfluidic compartments, enabling analysis of their conformations and function within a native transcription-translation system.

## Introduction

Programming artificial cells with single chromosomes is a frontier for bottom-up synthetic biology. Chromosomes have been transplanted from one species to another^1^ and chemically synthesized and assembled from smaller fragments^2–5^, functioning as genomes for living cells. The *E. coli* chromosome is a ~ 4.6 megabase-pair contiguous polymer with a contour length of a few millimeters, packed into a micrometer-sized cell. The long DNA molecules dramatically expand when released from cells^6,7^. Active and passive processes condense the long DNA molecule into 3-D organizations^8–10^, compact yet amenable to dynamic opening processes^11–15^, but the direct link between physical properties and DNA transactions remains an outstanding question. Embedding *E. coli* chromosomes in semi-open synthetic compartments could advance our understanding by releasing the chromosome from its confined volume and/or complex, intertwined dynamics in vivo. Further engineering, with the required knowledge of such fundamental processes, could establish chromosomes as biologically active genomes for future autonomous artificial cells^16^.

Single-molecule studies in vitro have advanced our understanding of isolated DNA transactions with relatively short DNA molecules^17–24^, whereas engineering an active system supporting a broad range of biochemical processes requires reconstituting the entire flow of genetic information at the genomic scale. Although powerful, current methods on cell-free chromosomes using closed settings^25–32^, such as encapsulated cell mimics, e.g., liposomes, pose challenges for imaging, external manipulations, and exchange of solutions. The two-step process of isolating and then embedding a chromosome also increases the risk of fragmenting such long DNA molecules. It is therefore a major goal to integrate individual chromosomes in in vitro setups supporting steady-state cell-free gene expression, real-time observation of low-abundance proteins, and simultaneous monitoring of biological machines active on the DNA.

Here, we present a methodology to trap *E. coli* cells in flat, quasi-2D, semi-open compartments integrated into a multifunctional microfluidic chip that supports steady-state cell-free transcription and translation (TxTl). The trapped *E. coli* cells are gently lysed in situ, resulting in the transplantation of their chromosomes into the compartment. We apply an electric field to gently pull and stretch the chromosome into a thin capillary using an electric field and identify stable, blob-like structures along chromosomes. Fluorescent labeling of DNA and DNA-bound proteins from the donor cell enabled spatial mapping of genome-wide protein occupancies, including nucleoid-associated proteins, RNA polymerases (RNAP), ribosomes, and bacterial condensin clusters, in relation to the DNA blobs. Upon introducing the cell-free TxTl system, we measure the transcription activity by an RNAP dissociation assay and monitor stochastic protein synthesis from a single gene encoded on the genome. We next transition to studying the chromosome conformations as native proteins dissociate from DNA in the TxTl system, and then, when all proteins are nonspecifically degraded. We find that in the absence of condensins, the local DNA blobs are removed and expand on the genome scale. Finally, we study the chromosome conformational changes in response to molecular crowding in the TxTl system in comparison to buffer conditions. We observe a global compaction due to the background of endogenous *E. coli* proteins in the TxTl system, which is counter-balanced by swelling due to active transcription on the chromosome.

## Results

### A single native *E. coli* chromosome embedded in a semi-open compartment

**Fig. 1:**
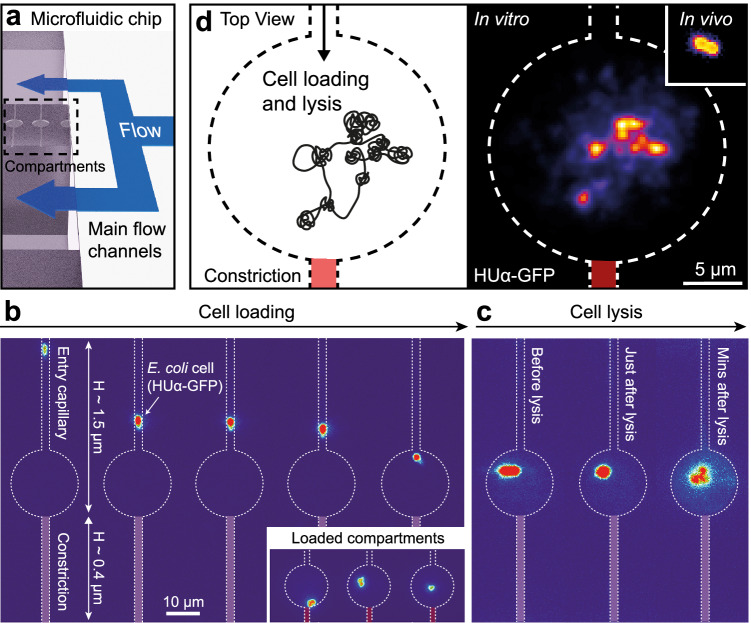
A single *E. coli* chromosome embedded into a semi-open compartment. **a** A 3-D illustration of the microfluidic chip with semi-open compartments flanked by two main flow channels for introducing cells, various buffers, and a cell-free transcription-translation system. **b** Fluorescence time-lapse montage of an *E. coli* cell loaded into the compartment and imaged by HUɑ-GFP expressed from a plasmid in the bacterium. From left to right: Entry of the cell into the top capillary during centrifugation of the microfluidic chip (filled with a cell suspension in sucrose buffer), and its retention in the main chamber by the thin bottom constriction capillary. Inset: three compartments loaded, each with a single bacterium after centrifugation. **c** Three images tracking a bacterium upon exchange to lysis buffer: Left (before)—having a normal ellipsoidal shape, Center (during)—transformed into a spheroid; Right (minutes after)—a blob-like expanded structure. **d** Left: scheme of a transplanted chromosome after lysis in the compartment. Right: Fluorescence of an HUα-GFP labeled chromosome before (inset) and after lysis. The fluorescence image was deconvolved with Huygens v24.04 to highlight the blob-like structures. The scale bar applies to both images.

To study a native chromosome as it emerges from the cellular environment, we developed a methodology to transplant and trap a single chromosome in a synthetic semi-open compartment that enables exchange of materials by diffusion^33–36^. We constructed flat circular compartments of height 1.5 µm and diameter 20 µm flanked by two narrow capillaries connected to two main flow channels providing continuous fluid exchange (Fig. 1a and Supplementary Fig. 1). One capillary was of the same height as the compartment to allow entry of a single *E. coli* cell (h ~ 1 µm), whereas the second created a constriction with a smaller height of ~400 nm, for entrapping the cell inside.

We loaded into the main flow channels *E. coli* K-12 MG1655 cells that were grown to the exponential phase and incubated with sucrose buffer as a required pre-conditioning step before cell lysis^6^. For tracking by fluorescence microscopy, cells contained a plasmid coding for the histone-like protein HUα, which binds the chromosome as a nucleoid-associated protein with no strong sequence specificity^37,38^, fused to green fluorescent protein (HUα-GFP). A single bacterium was then centrifuged from the upper flow channel into the compartment (Fig. 1b, Supplementary Fig. 2). Following on-chip cell wall degradation, we exchanged the buffer in the chip to cell lysis buffer, thereby gently embedding the chromosome into the compartment. Upon in situ cell lysis, the transplanted HUα-GFP labeled chromosome rapidly detached from the interior of the cell (Fig. 1c), attaining an expanded blob-like structure within ~10 minutes (Fig. 1d).

### Mapping native proteins bound to chromosomes by electric-field DNA stretching

**Fig. 2:**
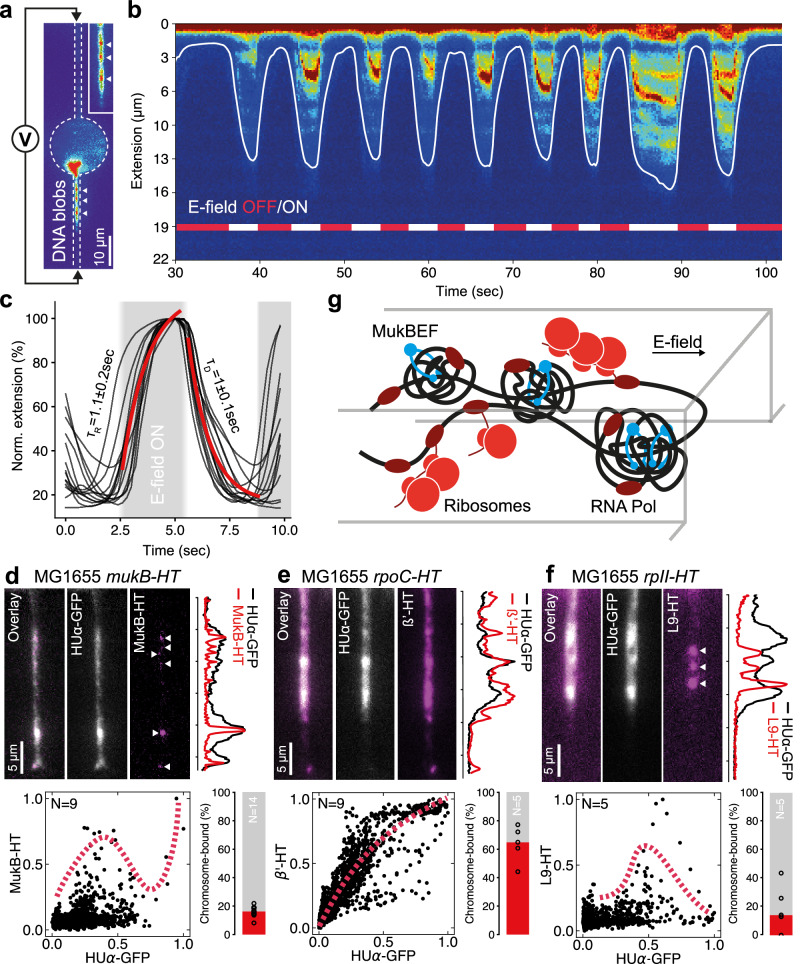
Electric field stretching of chromosomes and imaging native proteins bound to DNA. **a** Application of an electric field between two electrodes across a compartment loaded with a chromosome. Short chromosome segments pulled into the capillary revealed bright fluorescent HUα-GFP blobs (white arrowheads). **b** Repeated applications of the electric field (ON/OFF within a few seconds), and imaging of the HUα-GFP line profile along the lower capillary. The continuous white line indicates the DNA boundary detected by image processing. **c** Stretching and relaxing dynamics of the chromosome by repeated ON/OFF applications of the electric field (black lines), with a fit to mono-exponential rise and decay (red line). **d–f** (**Top**) Fluorescence snapshots of stretched segments from protein-bound chromosomes with a dual label of HUα-GFP and HaloTag (HT) fusions: **d** MukB (*mukB-HT* gene), **e** RNAP (*rpoC-HT* gene), and **f** ribosome (*rpII-HT* gene). Overlayed normalized dual color intensity profiles along the capillary on the right side of the images show: **d** spots of MukB aligned with DNA blobs (arrowheads), **e** uniform coverage of RNAPs on DNA, and **f** sparse bright spots of ribosomes excluded from DNA blobs (arrowheads). (**Bottom**) Scatter plot of an ensemble of measurements (N stretched chromosomes, as denoted) for each protein. The dashed red line is a guide to the eye. The fraction of chromosome-bound HT-fusion proteins is shown next to the scatter plot, computed from signals before and after lysis of single cells (N cells, as indicated in the bar graph and shown as black circles), with the red bar as median. **g** A sketch of the spatial organization of MukBEF, RNAP, and ribosomes, along the stretched bacterial chromosome, as deduced from **d**–**f**. Source data is provided as a Source Data file.

We then asked the following: Are the blob-like DNA structures stable? Do transplanted chromosomes retain their structural integrity during lysis, and are they decorated with native proteins bound to DNA within and outside the blobs? To test for major fragmentation of the chromosome and stability of the genome organization in the lysis buffer, we applied a weak alternating electric field across the compartment to pull and release chromosome regions into and from the thin capillary (Fig. 2a, b, and Movie S1). The HUα-labeled chromosome stretched and relaxed reversibly in about a second, consistent with relaxation times reported on mitotic DNA^39^, supporting the notion that the blobs were stable structures and that the chromosome was not fragmented on larger scales (Fig. 2c, Supplementary Fig. 3a).

We employed this stretching approach to spatially resolve bound proteins on the chromosome. We fused three proteins of interest to the fluorescent HaloTag (HT) reporter system^40^, each encoded on the chromosome in three separate *E. coli* strains (Methods): (i) MukB-HT—the bacterial condensin complex MukBEF, proposed as active loop extruder and key genome organizer that localizes across the chromosome as discrete clusters^41–45^, each consisting of ~36 MukB proteins^46^ that entrap DNA locally. (ii) β’-HT—one of the protein subunits of the RNA polymerase (RNAP). (iii) L9-HT—one of the ribosomal proteins. By measuring the HT signal before and after cell lysis with these strains, we could estimate the occupancy of the MukB, RNAP, and ribosomes on the chromosome to be about 15%, 65% and 10%, respectively (Fig. 2d-f).

The stretching experiments revealed striking fluorescent protein patterns bound to the chromosome. Despite the loss of ~85% of the MukB-HT signal during cell lysis (Supplementary Fig. 2a-c), discrete spots of MukB co-localized to the HUα-GFP signal of the DNA blobs (Fig. 2d, Supplementary Fig. 3b), suggesting that MukB may be responsible for their formation. The low occupancy of MukB on the chromosome is consistent with in vivo experiments reporting a DNA-bound MukB fraction of ~20% with a short residence time of ~50 seconds for MukB on DNA^46^. The HT-labeled RNAP and HT-labeled ribosomes had markedly different signatures from MukB-HT (Fig. 2e, f, and Supplementary Fig. 3b). The RNAP covered the DNA along its entire contour, with a signal roughly linearly proportional to the DNA signal, both at the regions of blobs and elsewhere. We could not detect a ribosome signal on many of the stretched chromosomes, but a few chromosomes had localized, sparse bright foci of ribosomes that were excluded from the DNA blobs, as evidenced by the anti-correlation to the HUα-GFP signal. The exclusion of ribosomes from the compact DNA blobs possibly suggests large polysomes that were tethered to DNA by RNAP-mRNA complexes.

### Measuring protein synthesis from a single chromosome

**Fig. 3:**
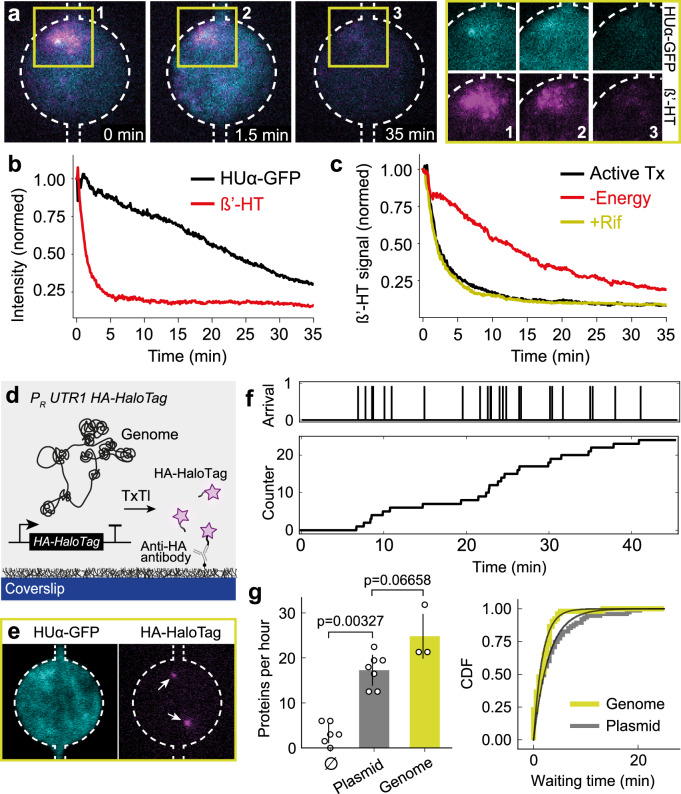
The rate of transcriptional elongation and of protein synthesis on a single chromosome. **a** Fluorescence time-lapse images of an *E. coli* chromosome in a compartment labeled by nucleoid-associated proteins (HUα-GFP, cyan) and RNAP (β’-HT, magenta). At t = 0 min, the lysis buffer was exchanged for a TxTl system. The decay of the signal is shown overlayed (left) and in separate (right) channels. **b** Decay of fluorescence signals of HUα-GFP and β’-HT from one chromosome upon introducing the TxTl system, reporting on the rate of transcriptional elongation, followed by decay and dilution out of the compartment. The dynamics were observed for three chromosomes. **c** Fluorescence signals of RNAP (β’-HT) upon introducing the TxTl system as in (**b**) for three conditions: active transcription (not blocked) (black curve), blocked transcription initiation by rifampicin (Rif, yellow curve), and depleted of energy buffer for transcription (red curve). The dynamics were observed with five chromosomes in at least two independent biological experiments for each condition. **d** Schematics for measuring the protein synthesis rate from a chromosome engineered with a HaloTag (HT) gene cassette. Nascent HA-tagged HaloTag proteins were captured through surface-immobilized Anti-HA antibodies for imaging. **e** Exemplary dual-color fluorescence image of a chromosome labeled with HUα-GFP, and two surface-captured HA-tagged HaloTag proteins (white arrows). **f** Time traces of arrival time and counts of HA-HaloTag protein spots captured and recorded on the surface during the TxTl reaction from a chromosome in the compartment. **g** The rate of HaloTag proteins expressed in a TxTl reaction with single plasmids, chromosomes, and without DNA (as a negative control). Data show independent replicates, and the bars with error bars (S.D.) represent their means. Two-sided Mann-Whitney U tests were performed to compute the statistical significance. The cumulative distributions of protein arrival times are shown with fits to mono-exponential distributions (black lines). The fits gave arrival rates of 0.67 ± 0.05 min^−1^ for chromosomes and 0.37 ± 0.01 min^−1^ for plasmids. The data were combined from independent experiments with plasmids (*N* = 7) and chromosomes (*N* = 3). Compartment diameter, 20 µm. The dashed white lines outline the compartments and capillaries connecting to the two main flow channels. Source data is provided as a Source Data file.

In the next set of experiments, we introduced an *E. coli* endogenous cell-free TxTl system with the aim of measuring the rate of protein synthesis from a single *E. coli* chromosome in the semi-open compartment. The stable and uniform binding of native RNAPs on the chromosome in buffer suggested that upon lysis, the RNAPs were caught in arrest of transcriptional elongation, most likely on the ~700 σ_70_-regulated housekeeping genes of the genome^47^. We asked whether the bound RNAPs maintained their functionality and could resume activity, and if so, use them to estimate the rate of mRNA transcription in the TxTl system. We introduced the TxTl system into the microfluidic chip, tracking the fluorescence signal of chromosome-bound native RNAPs. The RNAP signal decayed exponentially within a couple of minutes, much quicker than the HUα-GFP decay, suggesting a fast energy-driven release of the RNAPs from the DNA, followed by diffusion out of the compartment (Fig. 3a, b). To verify transcriptional activity, we used a TxTl system lacking the energy buffer containing nucleotides for transcription (Methods) and observed a slower release of native RNAPs from the chromosome, as expected (Fig. 3c). In addition, we repeated the experiment using a full TxTl system supplemented with 500 nM rifampicin, an inhibitor of transcription initiation, thereby blocking activity of endogenous RNAPs from the TxTl system (Supplementary Fig. 4)^48^ and preventing any competition with native RNAPs. Rifampicin did not change the decay in native RNAPs signal, therefore excluding competition effects and suggesting native RNAPs had completed transcribing mRNAs on the chromosome before diluting out of the compartment. Given the observed exponential timescale of ~2 min and a typical gene length of ~1000 nucleotides, we obtain an elongation rate of ~10 nucleotides per second, which agrees well with bulk measurements in the TxTl system^49^.

We next sought to directly measure the rate of cell-free protein synthesis in the compartment. We probed the typical activity of a gene by integrating a cassette on the chromosome with a strong *E. coli* promoter, ribosomal binding site, and HA-tagged HT protein (HA: Human influenza hemagglutinin tag). The HA-tag allowed the capture of synthesized HT proteins on the surface of anti-HA antibody functionalized compartments for imaging (Fig. 3d, Supplementary Fig. 5)^36,50,51^. Recording single-molecule HT appearances on the surface of the compartment, we observed an exponential waiting time distribution between protein synthesis events, with an approximate rate of one protein synthesized every 3 minutes, or ~20 proteins per hour from the gene on the chromosome. Interestingly, the same result was obtained using single shorter recombinant DNA molecules (plasmids) with the same cassette (Methods, Fig. 3e-g, Supplementary Figs. 5 and 6). This result contrasts with the amplification reported in bulk TxTl with rates of ~10^3^ protein per hour and DNA template^49^ but agrees with recent work on coupled TxTl from a single DNA molecule in a highly dilute limit^48^.

### Local chromosome compaction in the TxTl system by native condensin proteins

In the next set of experiments, we returned to the conformations of the chromosome in the compartment, but in a TxTl system rather than a buffer. Intrigued by the differences in dissociation dynamics of native HUα and RNAPs from the chromosome in the TxTl system (Fig. 3a-c), we sought to examine the stability of MukB proteins and their contribution to the conformation of the chromosome.

**Fig. 4:**
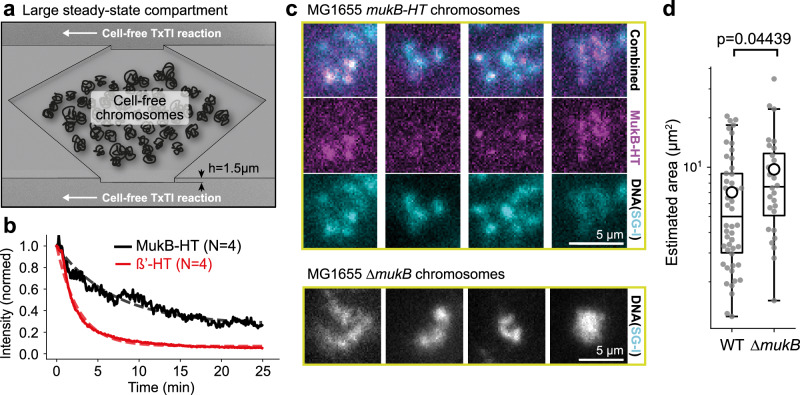
Compacted state of chromosome in a TxTl system maintained by native condensin MukB. **a** A 3-D schematic of multiple *E. coli* chromosomes (typically >10) transplanted into a large diamond-shaped compartment, flanked by two flow channels used to introduce *E. coli* cells, various buffers, and the cell-free transcription-translation (TxTl) system. The compartment is closed by a glass coverslip to assemble the microfluidic chip. **b** The fluorescence signals of MukB-HT and RNAP (β’-HT) in separate experiments, each averaged over 4 chromosomes from two independent biological experiments in large compartments. The dashed colored lines show fits to mono-exponential decays (*τ*_MukB-HT_ = 6.89 ± 0.20 min and *τ*_RNAP_ = 2.63 ± 0.03 min) with immobile fractions (A_MukB-HT_ = 0.27 ± 0.01 and A_RNAP_ = 0.071 ± 0.002). **c** Four exemplary snapshots of chromosomes extracted from *E. coli* strains with (upper row showing double-color images) and without (lower row showing grayscale images) a *mukB* gene transplanted into large compartments. For both cases, the DNA was labeled with SYBR Green I (SG-I). MukB-HT was labeled with MaP655-Halo before cell lysis. **d** The estimated area of chromosomes with and without the *mukB* gene after transplantation into large compartments and ~25-minute incubation in TxTl and SG-I. The boxplot extends from the first quartile (25%) to the third quartile (75%) of the data, with a line at the median and a circle at the mean. The whiskers extend from the box to the farthest data point lying within 1.5-fold the inter-quartile range from the box. Individual chromosomes are shown as the smaller gray points (*N*_WT_ = 67 and *N*_ΔmukB_ = 47), combined from two independent biological experiments. A two-sided Mann-Whitney U test was performed to compute the statistical significance between the two distributions. Source data is provided as a Source Data file.

Given the sparsity of MukB clusters on individual chromosomes (Fig. 2d), we increased the statistical ensemble by building a second microfluidic chip with larger, diamond-shaped compartments (100×70×1.5 µm³), each accommodating multiple chromosomes within a single microscope field of view (Fig. 4a, Supplementary Fig. 7a-c). After lysis of cells containing fluorescently labeled MukB-HT, we identified between 2-9 (median of 5, *N* = 5) chromosome-bound MukB clusters on single chromosomes (Movie S2, Supplementary Fig. 7b). We introduced a TxTl system supplemented with the intercalating DNA dye SYBR Green I (SG-I), replacing HUα-GFP as the DNA label because of its dissociation from DNA in the TxTl system (Fig. 3a, b). We observed a slow exponential decay of the MukB-HT signal with a time constant of ~7 min (versus ~3 min for RNAP in separate experiments), with ~30% remaining bound to the chromosome ( ~ 7% for RNAP) after 25 minutes (Fig. 4b, Movie S3). Despite minor cell-to-cell HT labeling heterogeneity (see Methods, Supplementary Fig. 8), we found ~90% of SG-I labeled DNA blobs colocalized with the ~30% MukB-HT spots (Fig. 4c, and Supplementary Fig. 7c), indicating the remaining chromosome-bound MukB maintained stable high-density DNA blobs on the chromosome in the TxTl system.

As a reference, we used *E. coli* chromosomes with a deleted *mukB* gene^43^, which appeared in the same TxTl conditions as a homogenous SG-I signal with fewer blob-like structures than for chromosomes with the *mukB* gene (Fig. 4c), and with a ~ 1.4-fold larger estimated mean area than that with the *mukB* gene (Methods, Fig. 4d). As a control, we verified the low bulk MukB concentrations in the TxTl system reported in previous studies^52^. This was done by preparing two TxTl systems, one prepared from the *E. coli* strain MG1655 mukB-HT and another one from an unmodified strain, measuring MukB-HT fluorescence signals only slightly above background fluorescence signals (Supplementary Fig. 9). Taken together, during ~30 mins of our cell-free experiments, a small fraction of native MukB proteins remained bound to the chromosomes and maintained the compacted conformation compared to MukB-depleted conditions in the TxTl system.

### Chromosome conformation affected by molecular crowding and transcription

**Fig. 5:**
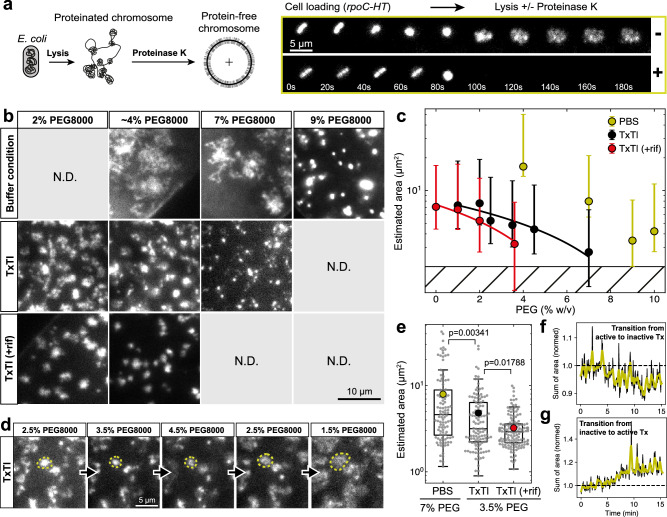
Effect of macromolecular crowding and transcription on conformation of protein-free chromosomes. **a** Scheme: protein-free chromosomes by on-chip cell lysis and protein degradation with protease Proteinase K (PKA). Right panels: exemplary time-lapse montages of *E. coli* cells labeled through RNAP (β’-HT) undergoing lysis (between 80-100 s) with (lower row) and without (upper row) protein degradation by PKA to obtain protein-free and protein-bound chromosomes, respectively. **b** Exemplary snapshots of SYBR Green I (SG-I) labeled chromosomes after transplantation and protein degradation with PKA at different PEG concentrations in dilute buffer, in a TxTl system, and in a TxTl system with 500 nM rifampicin (rif) to inhibit transcription initiation. **c** Chromosome areas were estimated in buffer conditions from 4 independent biological experiments at 4.0% PEG (*N* = 12 chromosomes analyzed), 7.0% (*N* = 105), 9.0% (*N* = 298), and 10.0% (*N* = 151). The TxTl system was analyzed from 3 independent biological experiments at 1.0% PEG (*N* = 321), 2.0% (*N* = 28), 2.5% (*N* = 122), 3.5% (*N *= 120), 4.5% (*N* = 173), and 7.0% (*N *= 90). The TxTl system with rif was analyzed from 2 independent biological experiments at 0.0% PEG (*N* = 146), 1.0% (*N* = 318), 2.0% (*N* = 184), and 3.6% (*N* = 139), where *N* is the total number of chromosomes analyzed. The graph shows the means of the population for the three different conditions, with error bars showing +/− 84.1% and 15.9% quantiles. **d** Exemplary snapshots of a region inside a large compartment populated with SG-I labeled chromosomes in a TxTl system undergoing a reversible transition from open (2.5% PEG) to compact (4.5% PEG) and back (1.5% PEG) with a change in PEG concentrations. Chromosomes were incubated with continuous imaging for ~20 minutes for each condition. The reversibility was checked in at least two independent biological experiments. **e** Estimated areas of chromosomes incubated in the three indicated conditions and labeled with SG-I (same data as in **e**). Individual chromosomes are shown as the smaller gray points. The boxplot extends from the first quartile (25%) to the third quartile (75%) of the data, with a line at the median and a circle at the mean. The whiskers extend from the box to the farthest data point lying within 1.5-fold the inter-quartile range from the box. Two-sided Mann-Whitney U tests were performed to compute the statistical significance between the distributions. The same statistics from **c** apply here. **f** The estimated summed area of all chromosomes in a large compartment was tracked during the transition from active to inactive TxTl (2% PEG), controlled with 500 nM rif (diluted from stock dissolved in DMSO). The black curve shows raw data after image processing, and the yellow curve shows data smoothed with a Gaussian kernel, both normalized by the initial area at the start of the experiment. The areas of 105 chromosomes were analyzed in an experiment. **g** Chromosomes from **g** were again exposed to a TxTl system (2% PEG) without rif (instead, DMSO was added at the same dilution level as in **g**). The overall chromosome area estimate is shown as the black curve and smoothed by a Gaussian kernel shown as the yellow curve, both normalized by the initial area at the start of the experiment. The areas of 123 chromosomes were analyzed in an experiment. Source data is provided as a Source Data file.

In the final set of experiments, we studied chromosome conformations without any native proteins transferred from the donor cell. For this, we added the unspecific protease (Proteinase K, PKA) to the lysis buffer. We confirmed protein degradation on the chromosome through the rapid fluorescent signal loss for RNAP-HT (Fig. 5a, Supplementary Fig. 10, and Movie S4 without PKA against Movie S5 with PKA), HUα-GFP, and MukB-HT (Supplementary Fig. 11, Movie S6). All three examples demonstrated that the deproteination of the chromosomes was effective for different types of DNA-binding proteins. We labeled the DNA using SG-I and measured the effective area of the chromosomes (Method) at different levels of the synthetic macromolecular crowding reagent polyethylene glycol (PEG) in dilute buffer conditions (as a reference) and the TxTl system (Fig. 5b).

The response with increasing PEG in dilute buffer revealed that chromosomes maintained an area of ~20 μm² at low PEG concentrations, which decreased with PEG and showed a sharp transition to the most compact conformation ( ~ 3 µm^2^) at 9% PEG without a further compaction at 10% PEG (Fig. 5c-e, buffer). We then measured the compaction level of the chromosomes with a TxTl system having a protein background concentration of ~25 mg ml^-1^^53^ and 2% PEG for optimal TxTl activity (Supplementary Fig. 4b)^54^. At low PEG concentration ( < 4%), the chromosomes were already ~2.5-fold more compacted than in the dilute buffer and attained the maximal level of compaction at 7% PEG, lower than in buffer (Fig. 5c, e), suggesting that background proteins in the TxTl system contributed to the chromosome compaction. Notably, the transition into compact µm-sized conformations with increasing PEG concentrations was moderate compared to the buffer conditions (Fig. 5c). The transition from open (at ~2.5% PEG) to compact (at ~4.5% PEG) conformations was reversible for more than 1 hour, with ~20 min incubation times for each condition to reach intermediate steady states (Fig. 5 d). The long-term reversibility further suggested that chromosomes maintained their integrity throughout the experiments, which might have been compromised by photo-induced DNA damage and residual nuclease activity in the TxTl system.

Strikingly, when adding rifampicin to inhibit transcription initiation, maximal compaction was obtained already at ~4% PEG, significantly lower than without rifampicin ( ~ 7% PEG), suggesting that transcription counteracts the macromolecular compaction beyond 1% PEG. We found that for 3.5% PEG, the estimated mean area of chromosomes with active TxTl was ~4.8 μm^2^ and ~3.2 μm^2^ for inactive TxTl, a ~ 1.5-fold difference in a statistically relevant manner. In addition, we found that chromosome compaction was reversible by transitioning from active to inactive and back again to active TxTl at 2% PEG (Fig. 5f, g, respectively). Taken together, we concluded that transplanted PKA-treated chromosomes were globally compacted by macromolecular crowding, induced by PEG and background proteins in TxTl systems, and slightly expanded by transcriptional activity, counteracting the macromolecular-induced compaction.

## Discussion

The semi-open compartments presented here offer means for rapid and multiple exchange of reagents for confinement of bacteria, in situ lysis, chromosome labeling, enzymatic degradation of native proteins, cell-free gene expression, and the response to molecular crowding. By design, the flow channels have low hydrodynamic resistance, whereas the thin capillaries are of high hydrodynamic resistance, which minimizes flow inside the volume of the compartment, setting diffusion as the main mechanism for transport of reagents into and out of the compartments^33^. We demonstrated genetically encoded labeling of just a few native proteins: condensins, RNAP, ribosome, and nucleoid-associated proteins. This suggests that other protein complexes might remain attached to transplanted chromosomes in their functional form, such as DNA replication. The interplay between physical confinement, DNA conformation, and gene expression at the scale of an entire chromosome raises interesting opportunities for future work.

The dose-response function of chromosome conformation with the synthetic crowder PEG is intriguing. The fact that a compaction transition in buffer occurs at an increased PEG concentration than in the TxTl system shows that ~25 mg ml^−1^ background proteins in the TxTl system suffice to compact the chromosome^52^. Adding rifampicin to inhibit transcription initiation pushed the compaction transition to lower PEG levels. Furthermore, the conformation of the chromosome was slightly expanded without rifampicin. These results suggest that transcription acting globally on the chromosome effectively swells the DNA, in possible agreement with recent simulations^55–57^. The general experimental approach presented here could be developed further to address this intriguing observation.

We inferred a reasonable transcriptional rate on the chromosome of ~10 nucleotides per second^49^, yet the overall TxTl rate of 20 proteins per hour and gene is ~50-fold lower than 10^3^ proteins per hour reported for bulk reactions in an endogenous *E. coli* TxTl system^49^. Interestingly, comparable low values were recently reported while observing the birth of individual proteins tethered to single DNA molecules via transient RNAP-mRNA-ribosome complexes^48^, together suggesting little to no translational amplification after the mRNA dissociated from DNA. This could potentially originate from short mRNA lifetimes and/or low translation rates at the single-DNA limit. Further exploration of physical compaction (e.g., compartment size, chromosome density) and biochemical activities (e.g., topoisomerase, RNase), toward functional life-like behavior outside a cell, may elucidate basic principles important in bacteria and possibly for the construction of future autonomous artificial cells.

## Methods

### Design, fabrication, and preparation of microfluidic chips

We designed the microfluidic chip layout with AutoCAD 2021 (Autodesk) and exposed 5” chrome masks (Nanofilm) with the MicroWriter ML 3 (Durham Magneto Optics Ltd). The mask was developed according to the manufacturer’s protocol. Next, a 4” silicon wafer (0.525 mm thickness, <100 > , p-type, University Wafers) was cleaned with acetone, isopropanol, and water. The wafer was cleaned with a plasma system (250 W, O_2_ at 40 sccm, 150 mTorr, 120 sec; March AP-300, Nordson). To promote adhesion of the SU-8 mold, the wafer surface was prepared with Hexamethyldisilazane (HMDS, Transene Company) for 30 s (after incubation, the wafer was dried by spinning for 30 sec at 3000 rpm), and a base layer from SU8 2000.1: 1) 7.5 sec at 500 rpm, 2) 30 sec at 1500 rpm with global UV exposure and hard bake. The first mold layer was SU-8 2000.5. The resist was spun on the wafer: 1) 7.5 sec at 500 rpm, 2) 30 sec at 1900 rpm. The resist was exposed with a mask and mask aligner (Karl Suss Ma6/BA6), baked, and developed using the manufacturer’s protocol. The second layer was SU-8 6001. The resist was spun on the wafer 1) 7.5 sec at 500 rpm and 2) 30 sec at 1500 rpm. The resist was aligned with the first layer, exposed, baked, and developed. The final layer was fabricated with SU-8 3050: 1) 7.5 sec at 300 rpm, 2) 30 sec at 2000 rpm. The resist was first exposed after aligning the first and second layers, and then baked and developed. For the plasmid experiments, the SU8 mold was constructed from only two layers, where the compartment and both capillaries were made from SU8 2000.5 and the flow channel from SU-8 3050. After verifying the SU-8 structures, the resist was hard-baked at 150 °C and slowly cooled to room temperature. The final dimensions were measured with a profilometer (Dektak, Bruker) and optical microscopy.

The wafer was placed in a petri dish and covered with ~40 mL of polydimethylsiloxane (10:1 PDMS:Curing agent, Sylgard 184, Dow Corning). The dish was placed for ~1 h under vacuum to remove bubbles and baked at 75-80 °C for ~4 h. The PDMS was cut into pieces using a scalpel and peeled off the wafer. Holes for the inlet and outlet were punched (0.75 mm diameter, Welltech Labs) on a cutting mat. The microfluidic chips were cleaned with isopropanol and blow-dried. The coverslips (#1.5, Marienfeld) were cleaned by boiling in 1) 96% ethanol for 10 min at 70 °C and 2) 3:1:1 H_2_O:NH_3_(25%):H_2_O_2_. The coverslips were blow-dried and stored in a dust-free box. Before bonding PDMS to the coverslip, we again cleaned the coverslips with the plasma system (250 W, O_2_ at 40 sccm, 150 mTorr, 120 sec). Then, the PDMS and coverslips were surface activated (100 W, O_2_ at 49 sccm, 180 mTorr, 20 sec) and brought in close contact for permanent bonding. The assembled microfluidic chips were baked at 75-80 °C for ~4 h.

The microfluidic chips were incubated with ~3 mg of photoactivatable silane polyethylene glycol (PEG)^58^ in 1.5 mL of anhydrous acetonitrile (Sigma-Aldrich) for ~30 min. After incubation, the microfluidic chips were successively washed with acetonitrile, an equimolar mix of acetonitrile and water, and water. The photo-sensitive PEG biochip was exposed with a UV cube (UV KUB), treated with 5 mg ml^−1^ NHS-Biotin (ThermoFisher Scientific) in 250 mM borate buffer (pH 8.6) for 20 min, and flushed with degassed 0.1% Tween-20 in PBS (phosphate-buffered saline, pH 7.4). The microfluidic chips were kept wet in a humid chamber until use (typically for 0-2 days).

### Defining the dimensions of small compartments in microfluidic chips

The lifetime of molecules in the compartment is quantified by the layout and diffusion coefficient $D$ of the molecule^33^

1 $$\tau=\frac{\pi {R}^{2}L}{2{DW}}$$

with $R$, $L$, and $W$ as the compartment’s radius, and the capillary’s length and width, respectively. To optimize the compartment layout, we initially constructed compartments with R = 5 µm, L = 40 µm, and W = 1.5 µm for single-molecule tracking experiments. We contained a single plasmid, tracked the plasmid position (pBEST HA-HT) with TrackMate^59^, and fitted the mean-square displacement to normal diffusion $\left\langle {r}^{2}\right\rangle=4{Dt}$ (Supplementary Fig. 6). We found a typical diffusion coefficient of D_D_ ~ 1.7 µm^2^ sec^−1^. We then designed a layout with a larger compartment size (R = 10 µm) to contain the *E. coli* chromosome for more than 1 hour and the plasmid for ~40 min, long enough for our expression experiments. The layout further gave a lifetime for mRNA τ_M_ around 7 min with a typical diffusion coefficient of D_M_ ~ 10 µm^2^ sec^−1^^60^, but was reduced to a few minutes (comparable to cells) through enzymatic degradation in the cell-free TxTl system^33^. The fast degradation presumably prevented mRNA from escaping the compartment. It allowed us to record the integrated translation activity throughout the mRNA’s entire lifetime by surface-capturing and imaging the nascent proteins.

### Experimental setup with single-molecule fluorescence microscope

Fluorescence images were acquired using Micro-Manager 2.0.0 and a custom-built single-molecule TIRF microscope, as previously described^36^. Briefly, tte lasers with 488 nm (100 mW, OBIS, Coherent) and 647 nm (120 mW, OBIS, Coherent) excitation were combined (DMSP605 and 5xBB1-E02, Thorlabs) via an objective mounted on an XYZ stage (MBT612D/M, Thorlabs) into a single-mode fiber (P5-460B-PCAPC-1, Thorlabs). The fiber was coupled into a mirror collimator (RC08FC-P01, Thorlabs) to expand the laser diameter to 8 mm, guided through an achromatic lens (f  =  150 mm, AC254-150-A-ML, Thorlabs), redirected by a mirror and filter cube (TRF59906, Chroma), and focused onto the back focal plane of the TIRF objective (60x, 1.49 NA, Nikon). The emission was focused with a tube lens (TTL200, Thorlabs) onto an EM-CCD (iXon Ultra 888, Andor Technology, Belfast, UK). The two lasers were controlled with an Arduino microcontroller (Arduino control software v1.2) and synchronized with the output trigger signal of the EM-CCD camera. The sample was translated in XY (Märzhäuser Wetzlar), and the TIRF objective was mounted with an in-house-built Delrin adapter for thermal insulation on a piezo stage to focus in Z (400 µm Fast PIFOC, PI). The objective was further enclosed with resistive heating foil (HT10K, Thorlabs) to set the temperature at 34 °C using a PID controller (TE-48-20, TE Technology). We constructed a custom stage chamber and flushed it with humid N_2_ to maintain a constant humidity and prevent the microfluidic chip from drying out. We acquired widefield fluorescence images, but the refractive index mismatch between PDMS and glass would also allow total-internal reflection fluorescence (TIRF) microscopy. Solutions were pulled from a 10 µL pipette tip attached to the inlet port through the microfluidic chip with a glass syringe (Gastight, Hamilton) and syringe pump (PHD Ultra, Harvard Apparatus).

The raw fluorescence images shown in the figures were processed by adjusting the minimum and maximum values to the same levels when comparing images in similar conditions using Fiji v1.0 software, and we mapped these 16-bit images into 8-bit for visualization on computer screens.

### Engineering the E. coli genome

We transformed *E. coli* K-12 MG1655 with the plasmid pSLTS (Forchheimer plasmid collection, Bacteriology and Genomic repository, Weizmann Institute) and induced the lambda red system for homologous recombination^61^. To generate the donor plasmid pKD4-HT, we transferred the previously established HaloTag reporter system with N-terminal fused HA tag (Supplementary Table 1)^48^ and the regulatory sites for transcription and translation from pBEST into pKD4^62^ (Forchheimer plasmid collection, Bacteriology and Genomic repository, Weizmann Institute) using PirPlus DH10β pir116 (Bacteriology and Genomic repository, Weizmann Institute) as cloning host. The plasmid was constructed by DNA assembly (NEBuilder HiFi DNA Assembly, NEB) of PCR fragments, amplified using KAPA HiFi HotStart ReadyMix (Kapa Biosystems, Roche), and verified through in-house sequencing.

The MG1655 strain with the induced lambda red system was transformed with the PCR product amplified (Supplementary Table 2) from pKD4-HT containing the HA-HT expression cassette, Kanamycin selection marker, and homologous regions. Several colonies were picked from agar plates with Ampicillin and Kanamycin for colony PCR with two flanking primers to verify the chromosomal insertion (Supplementary Table 2). PCR fragments with correct fragment lengths were sequenced. The final strain MG1655 P_R_ UTR1 HA-HaloTag was then cured from pSLTS by growing the cells at 37 °C on agar plates with Kanamycin. Growth curves for all strains were measured in a 24-well plate (Greiner) using a plate reader (ClarioStar Plus, BMG Labtech). A volume of 1.5 mL lysogeny broth (LB) medium was inoculated with 15 µL of overnight cultures (inoculated from −80 °C glycerol stocks) to measure the OD_600_ values every 3 minutes at 30 °C.

### Plasmid construction for HUα-GFP

The plasmid pBEST HUα-GFP was derived from P70a-UTR1-deGFP with Ampicillin resistance^63^. We amplified the gene *hupA* from *E. coli* K-12 MG1655 with colony PCR (KAPA HiFi HotStart ReadyMix, Kapa Biosystems, Roche) and primers (IDT, Supplementary Table 2). We fused the gene to the N-terminus of the *degfp* gene, separated by a short peptide linker (KRAPGTS). We assembled the PCR fragments with the NEBuilder HiFi DNA Assembly (NEB). Next, we reduced the transcription activity with a version of the PLlacO1 promoter (Supplementary Table 1) with PCR and primers, circularized the linear PCR fragment with a KLD Enzyme Mix (NEB), cloned it with chemically competent DH5α cells, verified the construct by sequencing, and transformed the final plasmid into the various MG1655 strains.

### Extraction of E. coli chromosomes

To extract the chromosome from *E. coli* cells, we followed the protocol by Pelletier et al.^6^. We grew the strains overnight, transferred the next day 100 µL into 10 mL fresh LB medium and Ampicillin to grow cells at 30 °C for ~2 hours, reaching the exponential phase (OD_600_ ~ 0.4). Because deleting *mukB* renders *E. coli* highly temperature-sensitive with defects in chromosome segregation^43^, we grew only the MG1655 *ΔmukB* strain overnight at ~22 °C to be directly processed without the second dilution and incubation step. We centrifuged 1.5 mL of cell suspension for 2.5 min at 5,000xg to harvest the cells. The supernatant was gently removed. The cells were washed in 500 µL PBS buffer and then resuspended in 500 µL of sucrose buffer (20 % sucrose (w/v), 100 mM NaCl, 10 mM EDTA, 10 mM NaPi, pH 7.3). The cells were centrifuged again and incubated with 2.5 µM MaP655-Halo^48^ (stock stored at −20 °C in dimethyl sulfoxide, DMSO, Sigma) in 100 µL sucrose buffer for 1 hour. The cells were gently mixed in a 2 mL tube with a rotisserie during incubation, followed by centrifuging and washing in 500 µL sucrose buffer.

For on-chip cell lysis, the plasmolyzed cells were harvested by centrifuging, resuspended in 75 µL sucrose buffer (for small compartments and for a high chromosome density in large compartments) or 750 µL sucrose buffer (for a low chromosome density in large compartments), and introduced into the compartments by centrifuging the microfluidic chip mounted on a tilted stage at a speed of 500×*g* for 10 minutes. Next, we introduced 300 µg mL^−1^ lysozyme from chicken egg white (Sigma-Aldrich) in sucrose buffer and incubated the chip for 1 h at room temperature to degrade the cell wall. We changed the buffer on the microscope-mounted chip at a flow rate of 1.5 µL min^−1^ with a hypotonic solution (100 mM NaCl, 0.5 mg ml^−1^ BSA, 20 mM HEPES, pH 7.5) to lyse the spherical cells. The flow rate was reduced to 0.5 µL min^−1^ after ~2 minutes of inflowing the lysis buffer on the chip. Chromosome-bound proteins were degraded by flushing a hypotonic solution supplemented with proteinase K (PKA, stock concentration of 800 units mL^−1^, NEB). The mix was prepared by adding 0.75 µL PKA to 50 µL hypotonic solution. After cell lysis and protein degradation with PKA, we first washed the microfluidic chip for ~25 mins with the solution that we used for the final experiments (0.5 µL min^−1^ with e.g., TxTl). We found the washing step to be critical for the removal of residual PKA and pre-conditioning of the microfluidic chip.

### In vivo HT labeling efficiency with MaP655-Halo

To test the in vivo labeling efficiency of the HaloTag with MaP655-Halo, we used the MG1655 *rpoC-HT* strain as a positive control for a highly and constantly expressed gene. As a negative control, we blocked the β’-HT with HaloTag Biotin Ligand (Cat. No G8282, Promega) before continuing with the standard HT labeling procedure: Bacteria in 100 µL sucrose buffer were first incubated with 2.5 µM HaloTag Biotin Ligand (stock stored at −20 °C in DMSO) for the negative control and 1 µL DMSO (stored at −20 °C) as the positive control. After a wash step with 500 µL sucrose buffer, the cells (positive and negative control) were incubated with MaP655-Halo as described above and imaged without cell lysis in large compartments. The HUɑ-GFP signal was used to segment bacteria trapped in large compartments and measure the β’-HT signals (Supplementary Fig. 8b). By measuring the average HT signal for hundreds of individual cells, we found a minor cell population in the positive control with low HT signals overlapping the HT signals of the negative control, suggesting that a small fraction of cells has a reduced labeling efficiency with the MaP655-Halo procedure.

### Local pulling of transplanted chromosomes with an electric field

After on-chip cell lysis, a ~ 5 V cm^−1^ electric field was manually applied across the compartment using a DC power supply (E3620A, Agilent) to pull the chromosome into the bottom capillary. Electrodes were constructed from standard electric copper wires and installed into a second pair of microfluidic in- and outlet ports. The fluorescence profile along the capillary was measured using Fiji v1.0 and plotted as a kymograph using the command “Reslice”. The kymograph was loaded in Python v3.7 and processed using Matplotlib v3.3, NumPy v1.20.3, SciPy v1.3, and scikit-image v0.17.2 (see processing software provided as Python Notebook in the Data Source). A further analysis of chromosome stretching and relaxation dynamics was not performed because of potential electroosmotic flows induced by the electric field inside the thin capillary. Future quantitative work needs to consider all physical aspects to derive a general mechanical chromosome model.

### Preparation of E. coli-based cell-free TxTl systems

The cell-free expression systems were prepared with *E. coli* (BL21 Rosetta 2) and K-12 MG1655 strains^63^. Briefly, bacteria were grown in 2xYT supplemented with phosphates. The bacteria were collected at an OD_600_  =  1.5–2, washed and lysed using a pressure cell. After the first centrifugation step (12,000×*g* for 10 min), the supernatant was incubated at 37 °C for 80 min for a run-off reaction. During the run-off reaction, we optionally added the HaloTag immobilization beads (Magne HaloTag beads, Promega) to pull down MukB-HT, providing a background reference to estimate the MukB-HT concentration in the cell-free expression system without pull-down. A bead volume of 100 µL was washed twice with S30B buffer (14 mM Mg-glutamate, 60 mM K-glutamate, 5 mM Tris, pH 8.2) following the manufacturer’s protocol, added to the run-off reaction, and later removed with a magnet. After a second centrifugation step at 12,000×*g* for 10 min, the cell lysate’s supernatant was dialyzed for 3 hours at 4 °C. After a final spin-down at 12,000×*g* for 10 min, the supernatant was aliquoted (29 µL) and stored at −80 °C. Before use, the cell-free TxTl system was thawed on ice, supplemented with the necessary solutions (10 mM Mg-glutamate, 80 mM K-glutamate, 4% PEG8000, 10.8 mg/ml Maltodextrin, amino acids, chemical energy buffer, and nuclease inhibitor GamS), filled to 78.3 µL with water, and gently mixed with a pipette. The 14x stock energy buffer solution contains 700 mM HEPES (pH 8), ATP 21 mM, GTP 21 mM, CTP 12.6 mM, UTP 12.6 mM, tRNA 2.8 mg/ml, CoA 3.64 mM, NAD 4.62 mM, cAMP 10.5 mM, folinic Acid 0.95 mM, Spermidine 14 mM, 3-PGA 420 mM. Finally, 9 µL of the cell-free expression system was mixed with 1 µL of water containing reagents such as a plasmid or a fluorogenic dye.

### Performing chromosome cell-free expression and buffer experiments in large compartments

Cells were lysed for 15 min under a flow of 1.5 µL min^−1^. The lysis buffer was exchanged under a flow of 0.5 µL min^−1^ by a TxTl system supplemented with 100-200 nM SYBR Green I (0.1-0.2x supplier recommended working concentration, Thermo Fisher Scientific, to get a 1:3 dye molecule to DNA ratio considering a dissociation constant K_D_ ~ 0.3 µM)^64^ for experiments in the large compartments, and, in the case of inhibited transcription, 500 nM rifampicin (15 µM stock solution was stored in DMSO at −20 °C). The chromosome experiments in diluted buffer conditions were performed under the identical flow conditions with PBS buffer supplemented with 5 mM Mg-glutamate and 4-10% (w/v) PEG8000 concentrations. We found that a small amount of PEG was required to visualize the chromosomes, consistent with previous observations^9,65^.

We omitted the energy buffer in preparing the cell-free TxTl system to check for reduced transcription activity on the chromosome in the compartments. Fluorescence images were acquired with an excitation power of 3 W cm^−2^ (488 nm) and 15 W cm^−2^ (647 nm), 20 ms exposure time, and 750 gain. Images were acquired every 2.5 seconds for alternating double-color imaging and every 5 seconds for single-color imaging.

To quantify the dissociation rate of MukB-HT and β’-HT from the transplanted chromosomes in large compartments after the inflow of TxTl, we measured the average intensity and background on several chromosomes and nearby free areas, respectively, using the Fiji software. The background levels were subtracted from the intensity levels, normalized by the initial intensity levels, and averaged over several chromosomes. The decay dynamics were fitted to $I=\left(1-A\right)\exp (-t/\tau )+A$, where *A* is the fraction of tightly bound proteins, *t* is the time during the experiment, and τ is the decay constant, using the non-linear least squares curve fitting implemented by SciPy v1.13.1.

### Image segmentation of chromosomes in large compartments

The cell-free chromosomes were segmented with the SYBR Green I using StarDist v0.9.1^66^ for estimating the chromosome areas. We used the pre-trained StarDist model “2d_versatile_fluo” for 2D to segment chromosomes in the large diamond-shaped compartments. We manually selected subregions inside the large compartments to exclude the fluorescence signal of non-lysed cells from the analysis. The detailed algorithm is provided as a Python Notebook in the Data Source.

For tracking the dynamics of chromosome conformations, we reduced the chromosome density (see the section for the extraction of *E. coli* chromosomes) and linked chromosomes when the segmentation overlaid with the segmentation from the previous image. The segmentation and tracking results were manually verified using Napari v0.5.1.

To find the fraction of MukB-HT spots on DNA blobs during the cell-free gene expression experiments, we localized and tracked MukB-HT spots and SG-I labeled DNA blobs on the chromosome using custom-written software^67^ (https://github.com/FerdinandGreiss/nanokit/). We deemed the two spots colocalized when MukB-HT and DNA blobs were closer than 0.32 µm (or 2 pixels).

### Functionalization of small compartments with antibodies

We followed the steps described by Vonshak et al.^50^. To functionalize the compartments with anti-HA antibodies, we mixed the biotinylated high-affinity (Roche Diagnostics, REF 12158167001, Clone 3F10, LOT 50271400) anti-HA antibodies (50 μg mL^–1^, ~500 nM, Sigma-Aldrich) at a 2:1 ratio with Streptavidin in PBS. We incubated the solution on ice for ~30 min. The antibodies conjugated with Streptavidin were incubated on the chip. After ~20 min, the main flow channel was thoroughly flushed with PBS to remove unbound antibodies.

### Measuring protein synthesis from chromosomes in small compartments

One hour after cell lysis and the transplantation of the chromosome into compartments, the solution in the microfluidic chip was carefully changed to PBS. The long waiting time allowed freely diffusing HT proteins to evacuate the compartments. The compartments were then functionalized with anti-HA antibodies, as described above. After carefully removing the unbound antibodies with PBS, we introduced the cell-free TxTl system and fluorogenic dye MaP655-Halo at a final concentration of 50 nM. We picked single compartments with intense HUα-GFP signals and acquired fluorescence images with an excitation power of 3 W cm^−2^ (488 nm) and 15 W cm^−2^ (647 nm) at a frame rate of 1 Hz (in alternating mode between the two wavelengths; the time delay between frames of a single excitation wavelength is therefore 2 secs), 100 ms exposure time, and 750 gain.

The fluorescent single-molecule spots were identified, localized, and tracked using custom software^67^ (https://github.com/FerdinandGreiss/nanokit). Furthermore, as only a few proteins were produced during an experiment, we linked single-molecule fluorescence spots with a clustering algorithm (scikit-learn v0.24.2, dbscan function) to reduce overcounting due to the temporal cut-off during the tracking process. Also, spurious spots outside compartments were removed from the analysis by manually outlining the compartments. The arrival times were pooled into a cumulative distribution function (CDF) to estimate characteristic arrival times with 1) a mono-exponential function $1-\exp (-t/\tau )$ with *t* as the waiting time and $\tau$ as the arrival time; and 2) a bi-exponential function $1-[A\exp (-t/{\tau }_{1})+(1-A)\exp (-t/{\tau }_{2})]$, where *t* is the waiting time, *A* the fraction of the population with arrival time ${\tau }_{1}$, and ${\tau }_{2}$ the arrival time of the second subpopulation. We found a mono-exponential function sufficient to describe the experimental data, suggesting a random Poissonian process. We fitted the experimental data to these functions using the non-linear least squares method implemented by SciPy v1.13.1.

### Performing single plasmid expression experiments in small compartments

The plasmid encoding the HA-HT gene was fluorescently labeled using a nick-translation protocol^48^. We labeled the plasmids with Atto647N as a far-red fluorophore to minimize the autofluorescence background from the cell-free TxTl system. Despite an overlap between the signals of the fluorogenic MaP655-Halo dye and plasmid, misidentification between protein and DNA was minimal due to the fast bleaching of DNA, on the order of a few minutes^48^. After incubating the antibody-functionalized microfluidic chip (prepared as described above) with 1 nM of fluorescently labeled plasmids for ~1 min, 10 µL of cell-free expression system and 50 nM fluorogenic dye were introduced to remove residual plasmids in the main flow channels and to initiate gene expression in the compartments. We picked compartments containing a single diffusing DNA molecule and started imaging. Fluorescence images were acquired with an excitation power of 15 W cm^−2^ (647 nm) and at a frame rate of 1 Hz, 100 ms exposure time, and 750 gain.

### Performing bulk TxTl experiments from plasmids

For a cell-free TxTl bulk reaction, 1 µL of a 10-fold stock solution (containing 10 nM pBEST P70a-UTR1-deGFP plasmid and 10-fold rifampicin or PEG8000) in water was mixed with 9 µL of TxTl system (for the PEG titration, PEG was omitted during the preparation of the TxTl system) and expressed at 34 °C in a 96-well plate (Costar) tightly sealed with a plastic lid (Cat. No. 3080, Costar). The fluorescence signals were recorded every 3 minutes using a plate reader (ClarioStar Plus, BMG Labtech).

### Statistics and reproducibility

No statistical method was used to predetermine the sample sizes. No data were excluded from the analyses. The experiments were not randomized. The investigators were not blinded to allocation during experiments and outcome assessment.

### Reporting summary

Further information on research design is available in the Nature Portfolio Reporting Summary linked to this article.

## Supplementary information

Supplementary Information

Description of Additional Supplementary Information

Supplementary Movie 1

Supplementary Movie 2

Supplementary Movie 3

Supplementary Movie 4

Supplementary Movie 5

Supplementary Movie 6

Reporting Summary

Transparent Peer Review file

## Source data

Source Data

## Data Availability

Data supporting the findings of this study are available in the article and its Supplementary Information section. The data generated in this study are provided in the Source Data file. Plasmids and *E. coli* strains are freely shared upon request from the corresponding authors. Source data are provided with this paper.

## Code availability

Custom Python code for processing the images is provided in the Source Data file. Custom code for single-molecule detection can be found on GitHub (https://github.com/FerdinandGreiss/nanokit/) and Zenodo^67^.
