# Supplementary material for: Bacterial chromosome conformation and cell-free gene expression in synthetic 2D compartments
Source: Nat Commun. 2025 Nov 14;16:10026. doi: 10.1038/s41467-025-65249-2 (PMC12618494; doi:10.1038/s41467-025-65249-2)
Supplement: Supplementary file 1 — Supplementary Information [file 41467_2025_65249_MOESM1_ESM.pdf]

Supplementary Information for

Bacterial chromosome conformation and cell-free gene expression in synthetic 2D compartments

Author list

Ferdinand Greiss[1,*], Shirley S. Daube[1], Vincent Noireaux[2], Roy Bar-Ziv[1,*]

Affiliations

[1]Department of Chemical and Biological Physics, Weizmann Institute of Science, Rehovot, 7610001, Israel

[2]School of Physics and Astronomy, University of Minnesota, Minneapolis, MN, 55455, USA

*Corresponding authors: F.G. (ferdinand.greiss@gmail.com), R.B.Z (roy.bar-ziv@weizmann.ac.il)

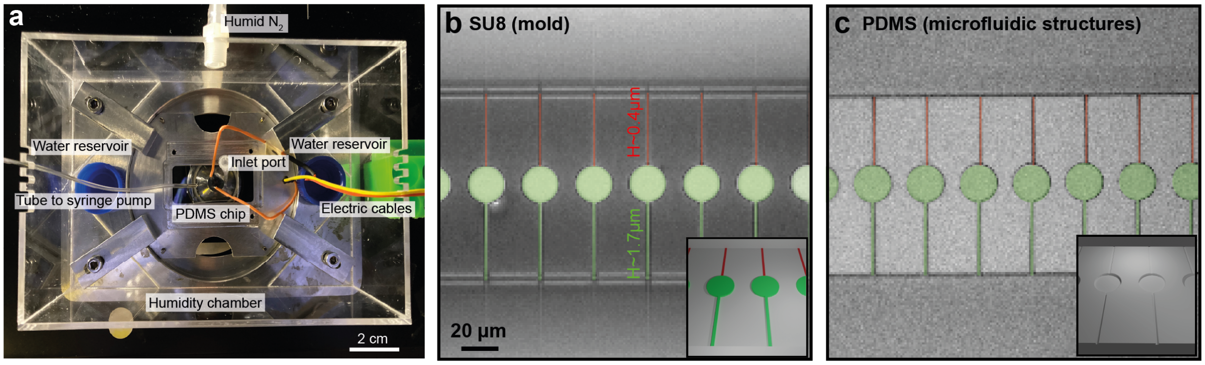

Supplementary Figure 1: Semi-open compartments to study individual cell-free chromosomes.
a) The experimental setup with a polydimethylsiloxane (PDMS)-based microfluidic chip integrated into a single-molecule fluorescence microscope, connected to electronics, and stabilized against environmental fluctuations through a custom humidity chamber. **b)** 3-layer SU8 mold with the first layer (colored in red) at a height of ~0.4 μm, the second layer (colored in green) at a height of ~1.5 μm, and the third layer at a height of ~75 μm. The third layer produced the two main flow channels. **c)** A brightfield image of the polydimethylsiloxane (PDMS) chip after baking the uncured elastomer on the SU8 mold.

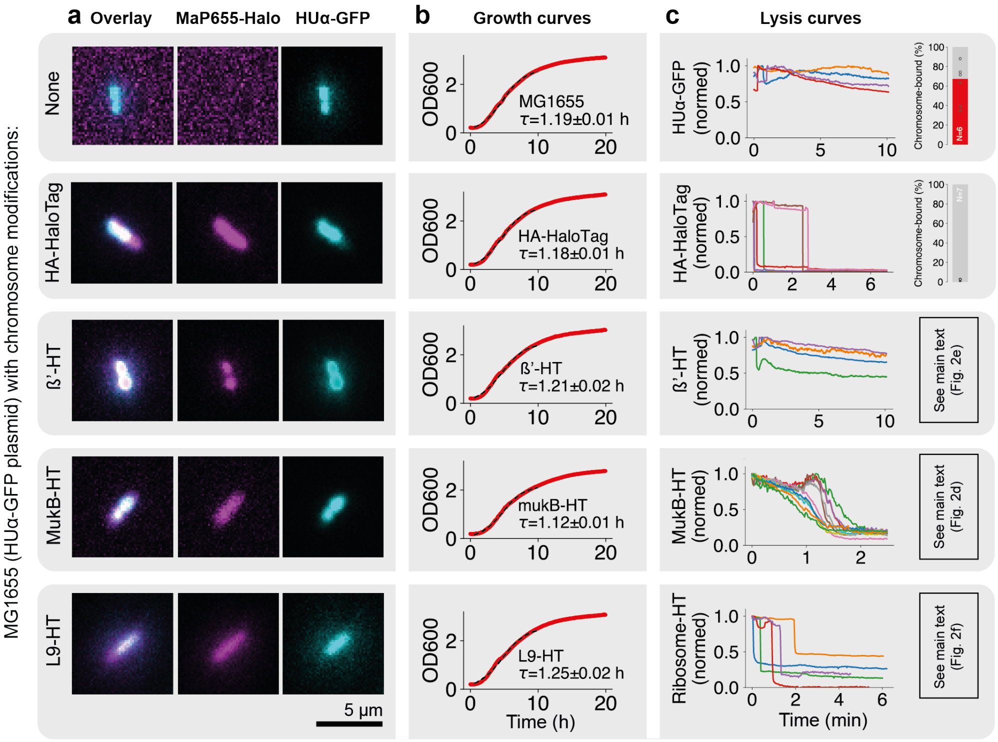

Supplementary Figure 2: The collection of bacterial strains with genes tagged with HaloTag and their growth curves and lysis properties.

a) All strains were derived from K-12 MG1655 with HUα-GFP expressed from plasmids and incubated with 2.5 µM fluorogenic dye MaP655-Halo before imaging. **b)** The growth curves were measured in a plate reader in lysogeny broth (LB) medium at 30 °C. The red curves were averaged from three replicates in a well plate and fitted to the logistic equation $OD_{600} = K/(1-(1-K/N_0)2^{-t/\tau}$ (shown as black dashed line), estimating the doubling times τ for each strain (indicated in the figure legend). **c)** The cell lysis curves were produced from single bacteria trapped in large compartments (N cells, as indicated in the bar graph and shown as black circles), with the red bar as median. We tracked the fluorescence signal and computed the ratios before and after cell lysis to find the fraction of chromosome-bound proteins. At least two biologically independent experiments were performed to produce the data. Source data is provided as a Source Data file.

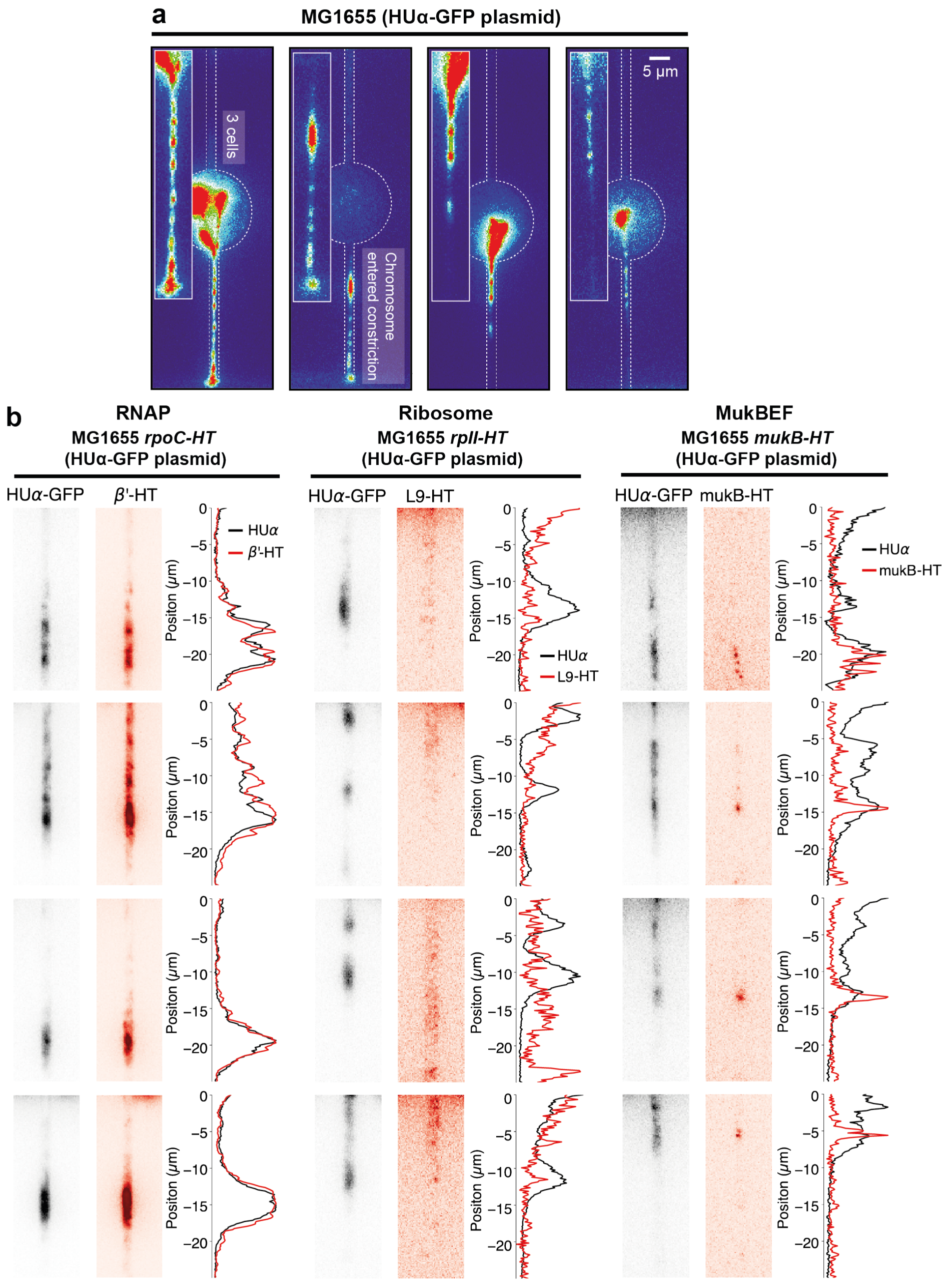

Supplementary Figure 3: Individual *E. coli* chromosomes stretched by an electric field.
a) The *E. coli* chromosomes were labeled with HUα-GFP, extracted *in situ*, and gently pulled into the lower capillary using a weak electric field. Here, four examples are shown, with one chromosome completely pulled into the small capillary (second panel from left) and another where three cells were lysed in a single compartment (leftmost panel). All cell-free chromosomes showed persistent bright HUα-GFP blobs along the stretched chromosome. **b)** Four further snapshot examples of stretched chromosomes with HT-labeled RNAP (β'-HT, left column), ribosome (L9-HT, center column), and MukBEF (MukB-HT, right column) in compartments using an electric field. Source data is provided as a Source Data file.

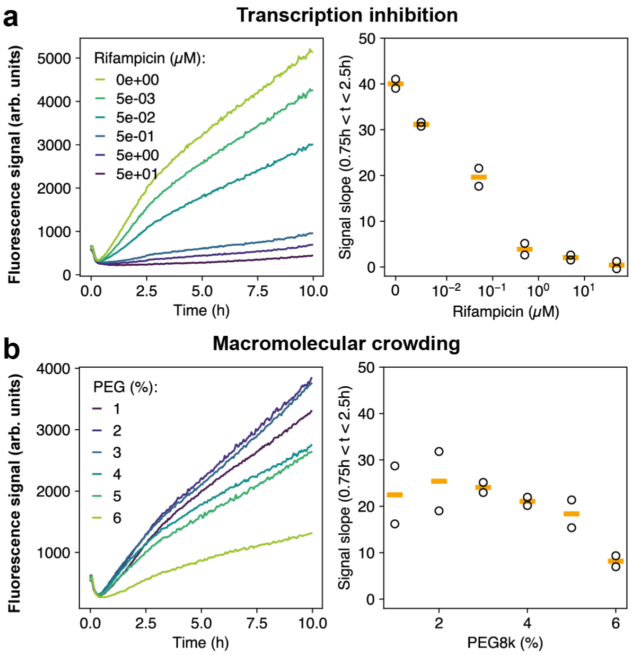

Supplementary Figure 4: Bulk cell-free gene expression with a titration of rifampicin and PEG.
a) Bulk cell-free transcription-translation (TxTl) dynamics of GFP synthesized from 1 nM plasmids and measured in 10 µL reaction volumes at 34 °C in a well plate using a microplate reader. We analyzed the TxTl activity by computing the slope of the fluorescence GFP signal at the beginning of the reaction (specific period indicated in the y-axis label in the right figures), where an unlimited amount of chemical energy for protein synthesis could be assumed. Later periods may show a slower protein synthesis because of already consumed chemical energy in the closed system. The yellow bars show mean values, and the black circles show data points from two replicates. We incubated the TxTl reaction with the indicated amounts of rifampicin to test the effects on the TxTl activity. **b)** Same as panel a, but with a titration of PEG8000 (% w/v). Source data is provided as a Source Data file.

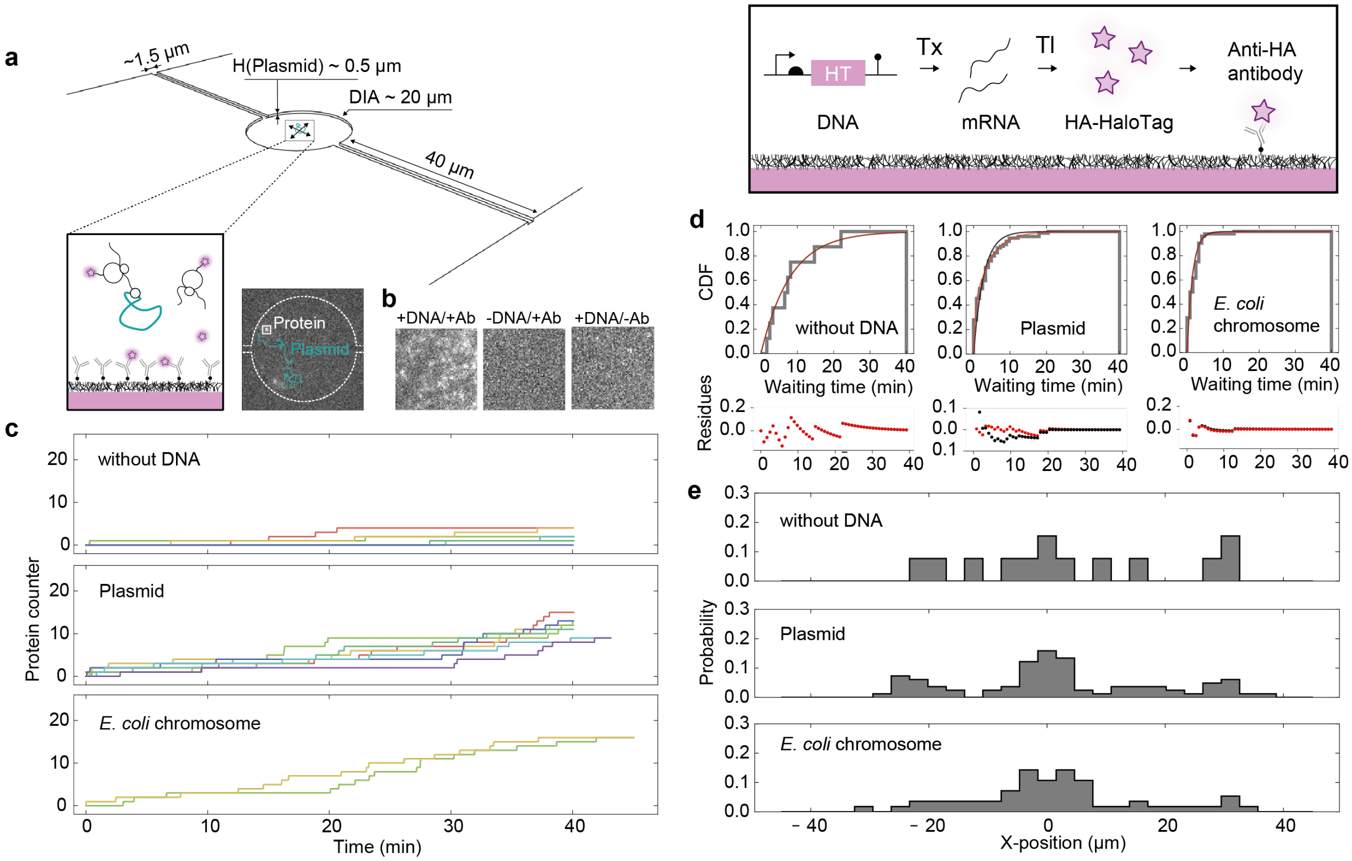

Supplementary Figure 5: The production and capture of HTs from single DNA molecules.
a) Schematic of a semi-open compartment with a freely diffusing plasmid encoding a cassette of native *E. coli* promoter, ribosomal-binding site, and HA-tagged HaloTag (HT) protein. RNAP transcribes the gene, and ribosomes translate the mRNA to produce HT proteins. The HA-HT proteins were captured on an anti-HA antibody-coated coverslip. A fluorescence image with the compartment (outlined with white dashed lines) containing diffusive (Atto647N-labeled plasmid) and static (surface-captured HT proteins) single-molecule spots. **b)** Fluorescence images with and without free-floating plasmid (encoding the HA-tagged HT cassette) in a cell-free TxTl system and 50 nM fluorogenic dye after 40 min of incubation on the microfluidic chip outside the compartments. As expected, only a few protein spots were detected when the DNA or anti-HA antibodies (Ab) were omitted from the surface. **c)** Protein counter during cell-free expression in different compartments without DNA (N=6), with plasmid (N=7), and with chromosomes (N=3). **d)** The cumulative distribution functions of protein arrival times in the compartments. The data were pooled from all experiments and fitted with a single (black curve) and double (red curve) exponential distribution to measure a potential deviation (residues are plotted in the lower panels) from random Poissonian arrivals, i.e., a single exponential distribution. **e)** Positional distribution of identified HT protein spots along the compartments. The capillaries were oriented along the X-axis. Source data is provided as a Source Data file.

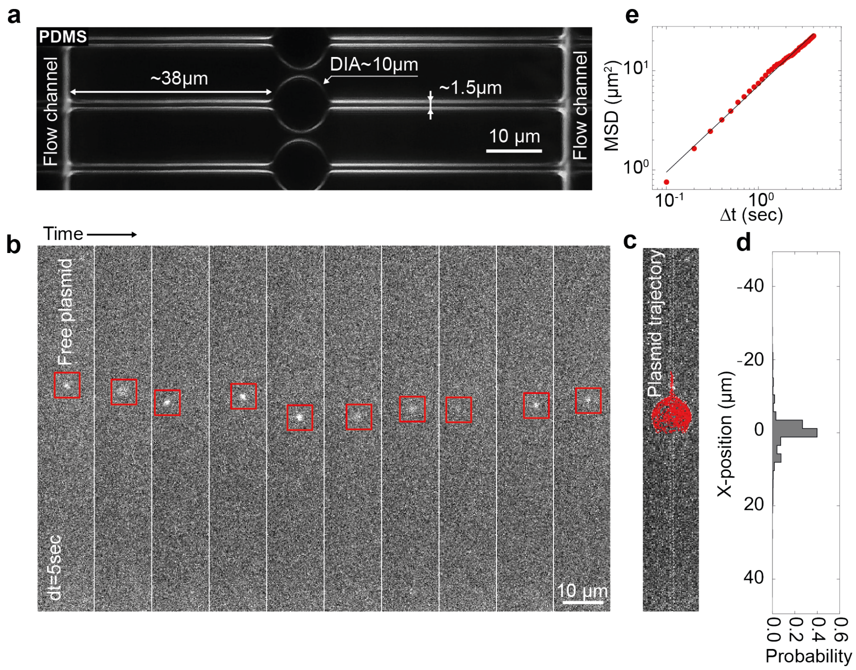

Supplementary Figure 6: Embedding short DNA molecules in semi-open compartments.
a) A darkfield image of compartments fabricated from polydimethylsiloxane (PDMS) to measure the diffusion coefficient of plasmids. Two capillaries connected compartments (diameter of 10 µm) with two main flow channels. The height of the compartment and capillaries was ~0.5 µm. **b)** Snapshots of a diffusing Atto647N-labeled plasmid in a compartment. **c)** The plasmid's trajectory (red line) was produced by single-molecule tracking. The dashed white line outlines the compartment. Plasmid diffusion experiments were reproduced in at least three experiments. **d)** The probability distribution of plasmid positions in the compartment and the capillaries. The compartment was centered at zero. **e)** The mean-square displacement (MSD) curve of a single plasmid (red points) and fit to normal diffusion (black line, D~1.7 µm^2 sec^{-1}). Source data is provided as a Source Data file.

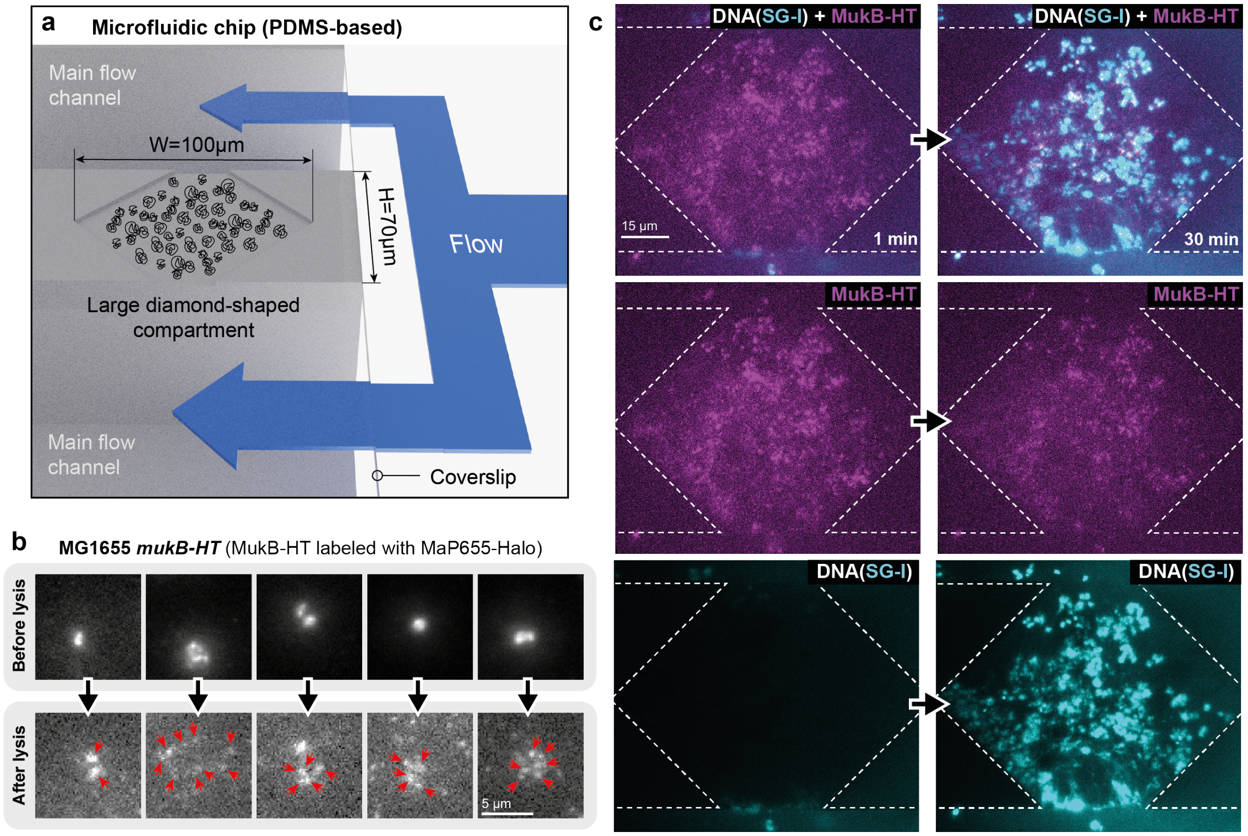

Supplementary Figure 7: Cell-free chromosomes with MukB-HT dynamics in large compartments.
a) A 3-D rendering of a large, diamond-shaped compartment for steady-state experiments, loaded with cell-free chromosomes and flanked by two main flow channels for the fluid exchange. **b)** Five examples of MG1655 *mukB-HT* chromosomes before (upper row) and after (lower row) cell lysis in large compartments with MukB-HT labeled with MaP655-Halo. The image brightness was adjusted for the upper and lower rows separately because of the difference in fluorescence intensity (~80% MukB is lost during cell lysis). **c)** Fluorescence images of a cell-free transcription-translation (TxTl) experiment with chromosomes extracted from MG1655 *mukB-HT*. MukB-HT was labeled before cell lysis with MaP655-Halo, showing clusters of MukB-HT proteins on cell-free chromosomes. The DNA intercalating dye SYBR Green I (SG-I) was flushed with the TxTl system into the microfluidic chip, labeling the DNA shortly after the start of the experiment. The results were repeated in two independent biological experiments.

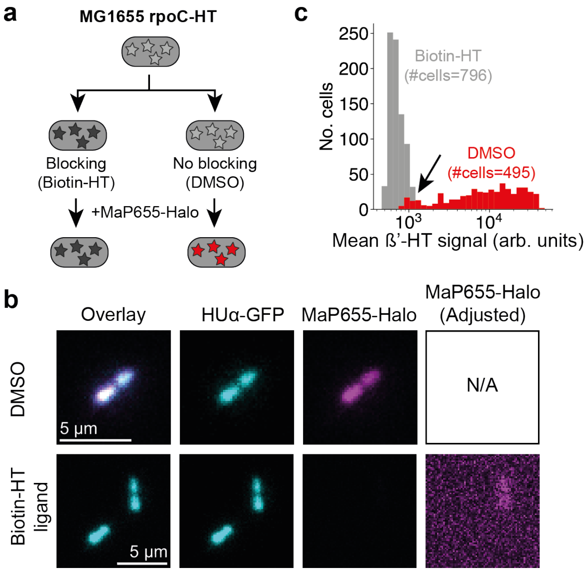

Supplementary Figure 8: Cell-to-cell HT protein labeling variability with MaP655-Halo.
a) A control HT-labeling assay with *E. coli* cells before cell lysis: Bacteria (*E. coli* MG1655 *rpoC-HT*) expressing HUα-GFP from plasmids were incubated with Biotin-HT (neg. control) and Dimethyl sulfoxide (DMSO). Both cell preparations were then incubated with the fluorogenic dye MaP655-Halo. **b)** The two double-color fluorescence image examples show single cells for the pos. control (one cell in the upper row) and neg. control (two cells in the lower row) after the treatment with MaP655-Halo. The brightness was adjusted for the negative control (lower row and rightmost column) to show only minor residual HT labeling (upper right cell) with prior blocking by the Biotin-HT ligand. **c)** Using this low HT signal reference, we could identify a minor peak around 10^3 arbitrary units in cells without blocking (DMSO; indicated by the black arrow), suggesting that a small ~10% cell subpopulation remained unlabeled with the MaP655-Halo labeling procedure. The HT-labeling was studied with RNAP proteins (β'-HT) as a highly expressed housekeeping protein from two independent biological experiments, producing the histograms. The number of analyzed cells is indicated in the figure legend. Source data is provided as a Source Data file.

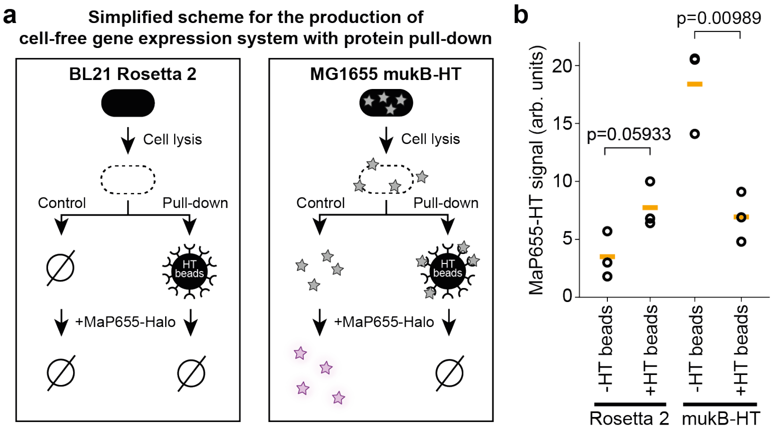

Supplementary Figure 9: Testing for residual MukB-HT from the cell-free TxTl system.

a) A schematic protocol for the pull-down of MukB proteins fused to HaloTag while preparing a cell-free TxTl system. We produced two TxTl systems by incubating with and without HaloTag immobilization beads (Magne HaloTag) during the run-off reaction. The TxTl systems were incubated with 50 nM fluorogenic dye MaP655-Halo to estimate the mukB-HT levels in BL21 Rosetta 2 (as a negative control) and MG1655 mukB-HT. **b)** The MaP655-Halo fluorescence signals in the TxTl systems were measured with a well plate reader in 10 µL volumes and after several hours of incubation at room temperature. The yellow bars show mean values, and the black circles show data points from three technical replicates. A two-sided T-test was performed for each, BL21 Rosetta 2 and MG1655 mukB-HT, to compute the statistical significance between the two distributions of HT pull-down and control. Source data is provided as a Source Data file.

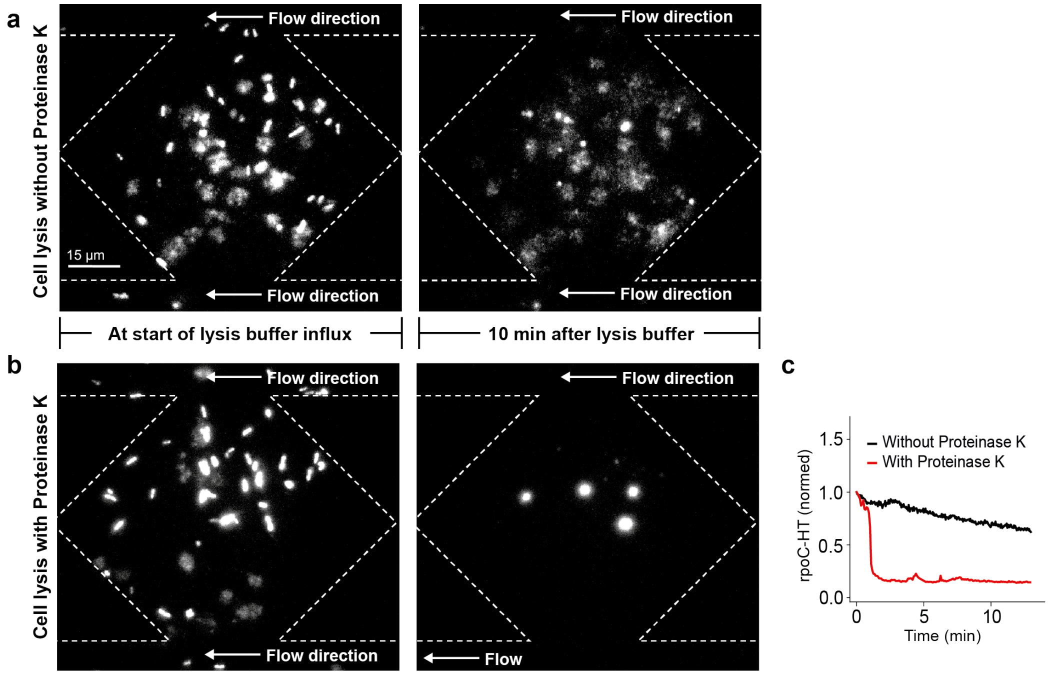

Supplementary Figure 10: Cell lysis and protein degradation in large compartments.
a) Representative fluorescence images of *E. coli* MG1655 (labeled with β'-HT) at the beginning of cell lysis and 10 minutes later without Proteinase K (PKA). **b)** Representative fluorescence images of *E. coli* K-12 MG1655 (labeled with β'-HT) at the beginning of cell lysis and after 10 minutes of cell lysis in the presence of PKA. Cells that were only later (>10 min) lysed or remained intact were imaged as discrete bright fluorescent objects. **c)** Fluorescence intensity traces (normalized by the initial fluorescence signal) of single cells labeled with β'-HT. PKA led to the fast decay in the β'-HT signal after cell lysis, suggesting a fast degradation of all RNAP proteins attached to the chromosome. Lysis without PKA maintains the chromosome-bound β'-HT. Cell lysis experiments with and without PKA were each repeated in at least three independent biological experiments. Source data is provided as a Source Data file.

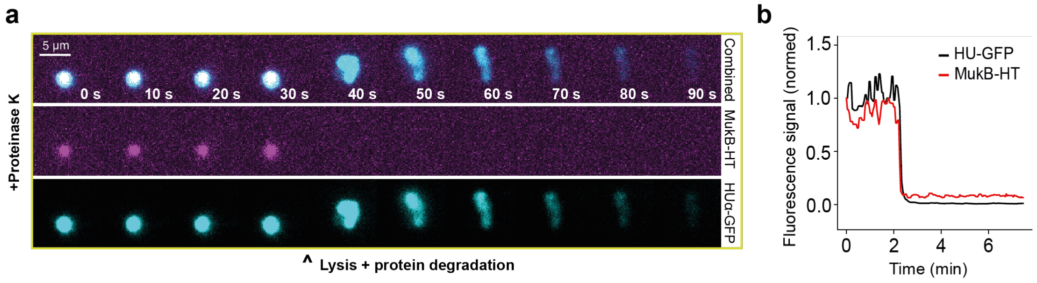

Supplementary Figure 11: Cell lysis and protein degradation dynamics of MukB and HUα proteins.
a) A fluorescence time-lapse montage with zoom on a cell expressing HUα-GFP from plasmids and MukB-HT from a MG1655 *mukB-HT* chromosome, undergoing lysis in the lysis buffer with proteinase K (PKA). **b)** Normalized fluorescence intensity traces of a single cell labeled with β'-HT (normalized by the initial fluorescence signal). PKA led to a fast drop of both fluorescent signals (HUα-GFP and MukB-HT) after cell lysis, suggesting a fast degradation of all proteins attached to the chromosome. The lysis without PKA maintains the chromosome-bound HUα-GFP and mukB-HT, as discussed in the main text. The cell lysis and protein degradation experiment was repeated in two biological replicates. Source data is provided as a Source Data file.

Supplementary Table 1: Partial plasmid sequences

**Name**	**Sequence (RBS is highlighted in violet)**
N-terminal HA tag for HT (HA tag highlighted with bold letters until HT's atg)	gcAATAATTTTGTTTAACTTTAAGAAGGAGATATAcc**ATGACCAGCTACCCATACGATGTTCCAGATTACGCTGGCCGCTTAATTAAACATATGACC**atg…
Promoter (small letters in front of RBS) and RBS region up to start codon for the HUα-GFP plasmid	gctgtgagcggataacattgacattgtgagcgggataacaagatactgagcacagctagcAATAATTTTGTTTAACTTTAAGAAGGAGATATAccatg…

Supplementary Table 2: Primers for *E. coli* genome engineering and plasmid construction

Name	Sequence (RBS is highlighted in violet)	Comments
MG1655-pKD4.f (capital letters = homolog region)	GTCAGTTTAAATTATAAAAATTGCCT GATACGCTGCGCTTATCAGGCCTAgaa agcaggtagcttgcagtg	Forward primer to generate PCR fragment from pKD4-HA_HT for insertion into the chromosome
MG1655-pKD4.r (capital letters = homolog region)	TCCTGCGCTTTGTTCATGCCGGATGC GGCTAATGTAGATCGCTGAACTTGcca tatgaatatcctccttagttcc	Reverse primer to generate PCR fragment from pKD4-HA_HT for insertion into the chromosome
HA_HT.chr.f	AAA TTG CCT GAT ACG CTG CG	Forward primer to verify chromosome integration of the HA_HT cassette and sequence
HA_HT.chr.r	GCA CCA GTA CGT TTT CCG CA	Reverse primer to verify chromosome integration of the HA_HT cassette and sequence
hupA.f	gcAATAATTTTGTTTAACTTTAAGAAG GAGATATAccatgaacaagactcaactgattgatg	Forward primer to lift the *hupA* gene from the *E. coli* genome and insert it into the pBEST backbone
hupA.r	AAGCTCCATGCTGGTCCCGGGAGCTC GCTTcttaactgcgtctttcagtgc	Reverse primer to lift the *hupA* gene from the *E. coli* genome and insert it into the pBEST backbone
