# Supplementary material for: Bacterial chromosome conformation and cell-free gene expression in synthetic 2D compartments
Source: Nat Commun. 2025 Nov 14;16:10026. doi: 10.1038/s41467-025-65249-2 (PMC12618494; doi:10.1038/s41467-025-65249-2)
Supplement: Supplementary file 2 — Description of Additional Supplementary Information [file 41467_2025_65249_MOESM2_ESM.pdf]

Description of Additional Supplementary Files

File Name: Supplementary Movie 1
Description: Reversible stretching of a bacterial chromosome (labeled with HUα-GFP) transplanted into a small cell-like compartment (after lysis), imaged during alternating electric field application in cell lysis buffer. Chromosomes were derived from donor E. coli expressing HUα-GFP from plasmids. The time stamp and scale bar are shown in the movie.

File Name: Supplementary Movie 2
Description: Lysis of a single E. coli MG1655 mukB-HT cell in a large compartment. Lysis was triggered by replacing the sucrose buffer with the cell lysis buffer. Chromosome detachment from the cell interior revealed discrete, chromosome-bound MukB-HT clusters, visible as bright foci. The time stamp and scale bar are shown in the movie.

File Name: Supplementary Movie 3
Description: Double-color time-lapse of transplanted chromosomes (after cell lysis) and native MukB-HT during transition from cell lysis buffer to a cellfree expression reaction containing 200 nM SYBR Green I. Chromosomes (SYBR signal, cyan) and MukB-HT (fluorescent HaloTag signal, magenta) were derived from MG1655 mukB-HT donor cells. The movie shows a small region in a large steady-state compartment. The time stamp and scale bar are shown in the movie.

File Name: Supplementary Movie 4
Description: Lysis of MG1655 rpoC-HT cells in a large steady-state compartment by buffer exchange from sucrose to lysis buffer. Chromosome-bound RNAP (β'-HT, fluorescently labeled) remained stably bound to the DNA after lysis. The time stamp and scale bar are shown in the movie.

File Name: Supplementary Movie 5
Description: Rapid RNAP (β'-HT) degradation during cell lysis in the presence of Proteinase K (PKA) added to the lysis buffer. A sucrose-to-lysis buffer exchange induced lysis of MG1655 rpoC-HT cells in a steady-state compartment. The fluorescent RNAP signal disappeared within a few seconds, indicating rapid degradation by PKA. The time stamp and scale bar are shown in the movie.

File Name: Supplementary Movie 6
Description: Double-color movie of MG1655 mukB-HT (magenta) and HUα-GFP (cyan, from plasmids)- expressing cells lysed in a steady-state compartment with Proteinase K (PKA) supplemented in the lysis buffer. The fluorescent MukB and HUα-GFP signals disappeared within a few seconds, indicating their rapid degradation by PKA. Three donor cells remained intact until the end of the movie (visible by their retained fluorescence). The time stamp and scale bar are shown in the movie.
