# Supplementary figures and images for: Bacterial chromosome conformation and cell-free gene expression in synthetic 2D compartments
Source: Nat Commun. 2025 Nov 14;16:10026. doi: 10.1038/s41467-025-65249-2 (PMC12618494; doi:10.1038/s41467-025-65249-2)

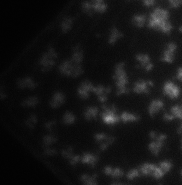

Supplement: Supplementary file 11 — Source Data [file 41467_2025_65249_MOESM11_ESM.zip › source-data/image-segmentation/exemplary-chromosome-image.tif]

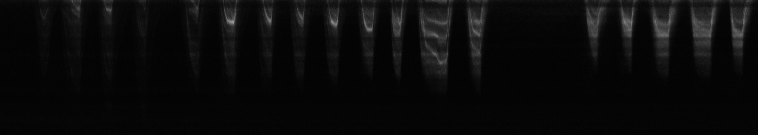

Supplement: Supplementary file 11 — Source Data [file 41467_2025_65249_MOESM11_ESM.zip › source-data/image-segmentation/full-kymograph-Fig2B.tif]
