# Supplementary material for: Bacterial chromosome conformation and cell-free gene expression in synthetic 2D compartments
Source: Nat Commun. 2025 Nov 14;16:10026. doi: 10.1038/s41467-025-65249-2 (PMC12618494; doi:10.1038/s41467-025-65249-2)
Supplement: Supplementary file 10 — Transparent Peer Review file [file 41467_2025_65249_MOESM10_ESM.pdf]

nature portfolio

Peer Review File

Bacterial chromosome conformation and cell-free gene expression in synthetic 2D compartments

Corresponding Author: Professor Roy Bar-Ziv

This file contains all reviewer reports in order by version, followed by all author rebuttals in order by version.

A version of this paper was originally rejected for publication by Nature Communications, however that decision was reconsidered after appeal by the authors.

Version 0:

Reviewer comments:

Reviewer #1

(Remarks to the Author)
In this paper, Greiss et al. report the extraction method of bacterial genomic DNA into the cellular-sized space by improving the artificial cell device constructed by their group (ref. 40). By the method, they extracted bacterial genome from single-cell bacteria trapped in the device and show its sensitivity to electric field manipulation and its ability of transcription and translation from the genome. I believe that the direction of this study is intriguing. However, at least from the present manuscript, the novelty and significance required in this journal are not readily apparent. Additionally, there are several concerns including problems in the references to prior research.

Novelty:
The foundation of the artificial cell device has already been reported by the authors (ref. 40).
The encapsulation of bacterial genome DNA in cell-size space has been achieved (ref 36).
An example of extracting and analyzing bacterial genomic DNA within a PDMS device has been provided (ref. 38).
The transcription and translation of bacterial genomic DNA in cellular-sized space are also described in ref. 36.
A previous study fusing Escherichia coli in cell-size space by microfabrication, which also show transcription and translation occur in the device, albeit it was from plasmids. This reference is missing (10.1038/s41598-018-30231-0).

Certainly, the confinement of a single E. coli in a semi-permeable space (artificial cell device) utilizing the slow diffusion rate is a first example. The issue, however, lies in the novelty of the analyses and discoveries made using this method, which remains unclear.

Significance:
In the last sentence of Discussion, there is a contribution towards simple tools for the bottom-up synthesis of self-replicating artificial cells. This is the case if the artificial cell space is made of biological molecules like liposomes. However, the space in this study is PDMS passivated with PEG. This device seems to be not self-replicated by biological activities, including genomic transcription and translation.

Other Issues:
The definition stating that the genome is intact or most likely extracted in an intact form is ambiguous. Even with ten 12kbp repeats, this only amounts to 100kbp, which is merely 1/40th of the E. coli genome. Moreover, at this level of fragmentation, there should be no impact on transcription and translation. The presumed state of being intact, as implied by the term "intact," is not shown in this manuscript.

The presence of GamS in the cell-free transcription-translation system is questioned. GamS protects degradation of linear DNA (ref. 9 or related literature). This contradicts the claim (line 137) that linear DNA is unsuitable.

To confirm genome integration, it is common to use primers attaching outside the insertion site of genome. By using the same primer attachment site for genome integration, it is impossible to distinguish whether the cassette was inserted into genome at the intended position, or if the plasmid has not been lost, etc. Therefore, at this stage, it is not proven that

transcription and translation have occurred from the genome.

The expression from the plasmid at a rate of 15 molecules per hour seems low. The reason for this is not explained. Additionally, the number of plasmids (or its estimation) present in the device is not described.

Polyethylene glycol (PEG), a high-molecular-weight polymer, is known to compact large DNA molecules like genomes (There are many reports. For example, ref. 38). However, the genome with cell-free transcription mixture is a relaxed form. It should be discussed why this difference occurred.

There is no discussion comparing these results with those from references 36 and 38.

The discussion section has numerous issues:
- Lack of citation of references, for example, in the second sentence and sentences 188-189.
- Unclear logic in the third sentence of the first paragraph and the first sentence of the second paragraph.
- Insufficient demonstration of the description in sentences 189 to 191.
- In the first paragraph, the last sentence mentions a Poissonian process. However, it is unclear how this is relevant to the interpretation of the results.

Reviewer #2

(Remarks to the Author)
The authors present an on-chip workflow to extract and stretch bacterial chromosomes by an electric field, and to perform cell-free protein synthesis. Fluorescence imaging combined with surface immobilization indicated that about 20 proteins were expressed per hour.

My main concern is that the authors put this study under the framework of ‘artificial cell’, which is misleading. They abuse terms like ‘on-chip artificial cells’, ‘microfluidic-based artificial cells’, ‘toward the construction of self-replicating artificial cells’, whereas in my opinion the main merit of this study is the biophysical characterization of in situ extracted chromosomes. The actual contribution ‘toward the construction of autonomous systems’ is more than questionable. The work should be repositioned to better fit what the authors have actually achieved.

Additionally, I think that more tests are needed to ascertain integrity and ‘near-native organization of the chromosome’ (page 7, line 186). Overall, the presented data set is too limited, and the conceptual and technical advances are not important enough for publication in Nature Communications.

Version 2:

Reviewer comments:

Reviewer #3

(Remarks to the Author)
This is a very interesting manuscript that describes a procedure for bacterial cell lysis in the confines of a flow cells followed by characterization of released chromosomes in response to transcription/translation and expression of MukB. The authors report that the chromosomes swell during transcription, condense in the presence of MukB, and that ribosomes localize away from the chromosomes. These results agree with previous conclusions that were obtained in live cells or on reconstituted much smaller systems. The main advantage of this system is that the studies are performed in situ on isolated chromosomes. This is a significant advance in the field that has many potential applications.

The weaknesses of the paper include poor organization, lack of clarity in the description of experiments, and insufficient statistical analysis. It is often unclear what was the purpose of experiments, what were the main conclusions and how they relate to the main thread of the paper. Experiments are often poorly described to the point that it is sometimes utterly unclear what has been done. For comparative studies, p-values must be calculated. The number of observed chromosomes is usually unreported. In some cases that are mentioned below, the analysis appeared questionable.

Specific comments.

1. Fig. 1 presents E. coli chromosomes isolated in flow cells. The size distribution of the chromosomes must be shown. Please compare the size or volume of the chromosomes with expectations based on at least some DNA models. A discussion of what limits the size of transplanted chromosomes would be appropriate. Please also compare sizes of chromosomes that were deproteinized or not.

2. The protocol for depoteinization of the chromosomes involves a treatment with Proteinase K. This protocol does not ensure complete removal of proteins and peptides from DNA. This needs to be reflected in the analysis.

3. Fig. 1b and 1f compare compartments with active (top half) and inhibited (bottom half) transcription. The comparison would be more instructive if the top and bottom parts of the figures showed the same compartment.

4. Line 738- indicate the extent of transcription inhibition by 500 nM rifampicin at the conditions of experiment. Please include a reference or show data.

5. Line 99 “we could observe chromosomes self-organizing into an intricate arrangement, likely due to steric hindrance by the neighboring chromosomes”. The shown data are insufficient to infer that the size of the chromosomes is dictated by the steric hindrance. A similar experiment at a reduced cell density would help evaluate the effect of the steric hindrance.

6. For all figures showing averages, how many chromosomes were quantified? Are the observed differences statistically significant?

7. Please provide details on quantification of SG-I intensity and its normalization. The question is especially important because chromosomes are often merged.

8. Fig. 1. SG-I intensity is a proxy for DNA accessibility and amount. It is not a good proxy for chromosome size. Please show the data for chromosome size.

9. Fig. S4. Please indicate which proteins were tagged in each experiment.

10. Fig. 3c Please mark the time periods when the electric field was applied.

11. Line 196. “Using the RNAP-labeled E. coli cells, the chromosome entered the capillary with the RNAP signal covering it continuously, apparently unaffected by the different DNA densities (Fig. 3e, Supplementary Fig. 5b)”. The statement sounds ambiguous, please rephrase. According to Fig. 3e, RNAP density is proportional to the HU density, not unaffected by it.

12. Fig. S6 needs diagrams of experiments or clearer explanations. Fig S6a- what happens between 1 min and 30 min? What is shown in Fig. S6c? How many chromosomes were analyzed and how did RNAP-HT make it into the graph given that it was not labeled for the experiment in Fig. S6ab.

13. Movie S4. What is shown on the three panels of the movie? This is a common problem with all movies, they need more extensive legends.

14. Line 235. Please describe the model in Fig 4d in a separate paragraph. This model integrates data described in multiple experiments presented in Fig 2-4.

15. How many MukB clusters did the authors observe in a chromosome?

16. Line 271, “Instead, we used the fluorescently labeled RNAP and ribosomes bound to the proteinated chromosome from the source bacteria”. This sentence is unclear, please rephrase. How was the experiment depicted in Fig 5 done?

17. The data in Fig 5a and 5b are very similar to those in Fig. 3e, 3f and arrive at the same conclusion. Please revise this section to avoid redundancy.

18. Line 276, “We introduced the cell-free gene expression system, tracking the expression dynamics through RNAP.” This sentence is too cryptic. Please explicitly state what was done. Was it a cell extract with RNAP or something else? Also, this sentence begins a description of a new experiment, put it into a new paragraph.
19. Line 283 “To gain further support, we omitted chemical energy from the reaction ...” What does this mean in practice?

20. The paragraph on lines 302-314 attempts to calculate the free energy generated by ongoing transcription in the isolated chromosomes. Please complete the argument by providing the calculated free energy. Please also keep in mind that RNAP can use up to 44% of the energy of nucleotide hydrolysis for mechanical work, but the actual number is often less than that.

21. The purpose of experiments in the section “Gene expression dynamics on a cell-free chromosome” is unclear. Please state the objective in the beginning of the section and conclusions in the end.

22. Supplemental figure 9c shows time lapse micrographs of diffusing “semi-conserved structures” in the chromosome. However, the conservation of these structures is limited to much shorter time intervals than the duration of the experiment. The placement of the asterisk in Fig. S9c seems arbitrary to this reviewer. This raises concerns about veracity of the entire experiment on diffusion rates.

23. Fig S9a outlines an experiment with MukB. However, this experiment is never mentioned in the main text of the article.

24. Neither objectives nor conclusions of section “DNA displacement fluctuations under cell-free gene expression” (lines 329-358) were clear to this reviewer.

25. Discussion restates the general knowledge in the field. Instead, it should highlight the main findings of the study and summarize how these findings enhance the state of the art.

Reviewer #4

(Remarks to the Author)
In this manuscript, Greiss and colleagues demonstrate that in vitro transcription can induce the swelling of E. coli chromosomes confined in diamond-shaped semi-open compartments when native binding proteins are degraded. They further show that chromosome-bound proteins can "protect" the chromosomes from debunching, leading to the formation of blob-like structures. The authors map the spatial distribution of condensin proteins, RNA polymerases, and ribosomes within the system and analyze gene expression dynamics on a cell-free chromosome. The concept of transcription-dependent chromosome swelling is new and interesting. Overall, I think the manuscript shows some important aspects in sufficient depth and is very worthwhile for considering publication in Nature Communications.

Concerns:
1. The authors describe chromosomes as active gels organized by molecular machines, but this claim appears speculative, as no direct experimental evidence is provided to support it. Similarly, the influence of macromolecular crowding on chromosome dynamics is not adequately addressed. While the authors state, “But without losing the expression activity, we could not directly test the response of DNA swelling by transcription to different crowding conditions,” it is worth noting that adding certain amounts of PEG (a crowding agent) to cell-free systems can enhance gene expression. The role of macromolecular crowding in chromosome dynamics should be further explored.

2. In Fig 1b, direct evidence of in vitro transcription occurring from the chromosomes is lacking. Without this, the functional relevance of rifampicin in the experiments remains unclear.

3. In Fig 3a, are HUα-labeled chromosomes in liquid or gel states? Why do they recover from the stretched state so quickly? The rapid recovery of chromosomes from the stretched state raises questions about the driving forces behind this process. Further clarification is needed.

4. The molecular mechanisms underlying transcription-induced chromosome swelling remain poorly understood, which is the weakest part of the manuscript. Elucidating these mechanisms would greatly strengthen the study.

Reviewer #5

(Remarks to the Author)
“Transcription-dependent swelling of a bacterial chromosome” presents an interesting and timely study at integrating some of arguably the most compelling intellectual and technological currents at the cutting edge of biotechnology broadly and synthetic biology specifically. The overall motivation for this work is irreproachable. The field requires guidance—at this stage, empirical or phenomenological will do—to understand and control the engineering implications of design choices around biomolecular interactions for confined genomes and cell-free expression systems.

However, the paper would benefit from major revision to clarify the main purpose and argument throughout the manuscript. The stated conclusions and speculated impact of the work are generally overreaching and should be reconsidered to more closely match the actual results of the study. The paper should be revised to clarify the argument and selection of the experimental studies in support of that argument. A main challenge is accomplishing this task lies in the highly multidisciplinary nature of the study, which puts tremendous responsibility on the authors to be thoughtful in their word choices and clear in their explication. Phrases like “single molecule” and “active gels” are used, without clear explanation or their relevance to the main argument of the work. With some care and attention, this paper can make a substantive contribution to the field. I recommend major revision prior to accepting for publication.

- What are the noteworthy results?

The observation of chromosome behavior confined to chip-based synthetic cells using cell-free expression systems is a welcome addition to the literature.

- Will the work be of significance to the field and related fields? How does it compare to the established literature? If the work is not original, please provide relevant references.

Yes.

- Does the work support the conclusions and claims, or is additional evidence needed?
- Are there any flaws in the data analysis, interpretation and conclusions?

See detailed comments below.

- Do these prohibit publication or require revision?

No, and yes, respectively. See detailed comments below.

- Is the methodology sound? Does the work meet the expected standards in your field?

Yes, the expectations of the field are generally met, except with regard to descriptions of the measurements and equipment

calibration. The image analysis, in particular, requires more detail to follow the progression from photons detected at a sensor to segmented images to reduced data quantities with associated measurement uncertainties.

- Is there enough detail provided in the methods for the work to be reproduced?

Yes, given that the cited references and resources are also consulted.

Detailed comments by manuscript line:

Line 20: “Autonomous biomaterials” seems to be a curious singular application area to highlight here. Why this example only, as opposed to others? What does “autonomous biomaterials” mean exactly here? Consider using clearer language.

Lines 22 to 23: Reaching the “single molecule limit” is a bold claim. Which single molecule? Imaging to resolve a single chromosome is decidedly more tractable than, say, a single protein. Or, is this meant to refer to a single fluor? Surely, this cannot be the case, given that the main claims of the paper pertain to chromosomal organization and conformation. The reader, however, cannot yet know this, and so I recommend clarifying this.

Lines 26 to 27: This sentence is confusing, because it seems to be attempting—with limited success—to convey too many disparate ideas. Consider breaking this into two separate sentences. The logic here is implicit and needs to be made explicit. This requires care and matters, because these ideas represent, I understand, the heart of the results presented in this paper.

Line 30: What is meant here by “nonequilibrium” exactly? In what way is the swelling “nonequilibrium”? The overall effect of the swelling can be amenable to an equilibrium description, even if the underlying molecular mechanisms that result in the emergent behavior of the swelling are the result of dynamic processes.

Line 32: The claim of “detailed balance locally broken” requires clarification. Even the “arrow” of time itself is not so at certain length and time scales. Similarly, detailed balance is a convenience applicable to its own certain length and time scales. It is unclear to which results this statement refers and how this statement relates to the main claims of the paper of transcription-dependent swelling.

Upon further reflection, perhaps a discussion on relevant time and length scales is missing. The paper seems to be aggressively forcing the conclusion of chromosomes as “active gels” for an undisclosed reason. This is all fine and good, but consider stating why this designation is edifying: does the fundamental formalism around active gels lead to new insight for synthetic biology or enable previously impossible engineering capability? The paper does not say.

Line 35: Consider removing “the” and replacing with “a”. Certainly, this is _a_ frontier but only one of many. Refrain from overstating the importance of this work. And, what is “bottom-up synthetic biology”? This term is not well-defined by the community. Consider revising for clarity. Is this meant to refer to so-called “bottom up” approaches to creating synthetic cells?

Line 42: The word choice of “transactions” reads strangely in English. Is this the best word here?

Line 44: This statement assumes that artificial cells will not confine chromosomes to a small volume. This need not be the case, and other researchers, as the authors are well aware, do indeed labor to create artificial cells of the same physical dimensions as extant, naturally-evolved cells.

Line 46: Consider clarifying for the reader what is meant here exactly by “active”. Physically active? Biologically active? Both? Something else entirely? It is unclear.

Line 49: Consider adding “biologically” before “active”. See comment for Line 46.

Line 53 to 54: This is such an important consideration. How was DNA breakage assayed and observed in this study? Or, what justifies its neglect?

Line 63: “High resolution” is meaningless in this sentence. Quantitatively, what resolution, or, better yet, resolution sufficient to resolve…what exactly?

Line 65: Check the verb tense.

Line 66: Is “energy source” the most correct and rigorous description for this? Perhaps “effective energy”?

Is it the transcription itself that is swelling the chromosome? Or, is the swelling the combined effect of many factors contributing to complex chromosomal dynamics, to wit: DNA melting and changes in conformation, entropy, and other associated polymer dynamics of the chromosome; electrostatic consequences of bound proteins that affect the solvent quality and associated polymer attributes related to conformation; effect of the physical confinement?

“Transcription” may be a catch-all phrase used by biologists for a biomolecular process, but using it to describe the physical phenomenon of counterbalanced forces acting to result in polymer behavior seems inappropriate and even, perhaps,

misleading. Rather, consider breaking down for the reader what underlying biomolecular dynamics and biophysics are meant here by “transcription”. Do not deliberately obfuscate matters for the reader.

Line 77: What is the dye molecule to DNA base pair ratio?

Page 3 Figure 1 panels b, d, f, and h: How were the raw micrographs altered in the preparation of the figure, if at all?

Line 100: What is the difference between the chromosomes distancing from each other due to “steric hinderance” versus, say, entropically-driven separation? Technically, “steric hinderance” refers to the effect of a molecule’s physical structure negatively impacting its ability to react chemically. Is this what is meant in this case? I doubt it. Consider using a more appropriate term.

Lines 106 to 107: Was the response of the microscope to increased fluorescence within a given area of the detector characterized for linearity within the range of intensities relevant to the studies presented?

Line 111: The use of “surprising” to begin this paragraph seems disingenuous. This seems unsurprising, especially given the reference. Was the compaction due to “transcription inhibition”? Or, was the compaction due to changes in the solvent quality for the chromosomal DNA due to the addition of rifampicin and its associated effects on molecules in the solvent? Is this behavior a transient that is then followed, as in cyto, by an expansion of the chromosome?

Line 130: This is perhaps an opportune moment in this review to inquire into how photobleaching, photonicking, and other photodamage to the DNA was mitigated and measured. I see that the devices are incubated in a nitrogen environment, the reason for which I do not believe has been given. Was this to reduce photodamage during imaging, assuming that the nitrogen passed through the PDMS of the device?

Furthermore, what is the surface chemistry of the devices? How much non-specific binding was observed, and how did it impact (or not) the fluorescence measurements or movement of the chromosome? What are the approximate Debye lengths on the surfaces, and how might that have impacted (or not) the diffusive exchange of components of the cell-free system on which some of the experiments rely?

Line 141: Is the deconvolution method given in the Methods? I see insufficient detail given in the Methods section.

Figure 3: Please indicate how the raw micrographs were processed to create the images included in the figure.

Line 183: This paper is an interesting and opportune amalgam of several disciplines, and not all readers are likely to be conversant in all of them. Consider stating what MukBEF is and why it was chosen, to help motivate its alleged significant contribution to determining the chromosome conformation.

Line 186: 20% of what? Please specify.

Line 238: I strongly recommend labeling this proposed model as “empirical” or “phenomenological”, because the model is not derived from first principles or even explicitly grounded in existing and relevant understanding in related fields, notably polymer physics. While the utility of this proposed model is unclear to me, others may find it useful in the future for qualitative guidance on chromosome behavior under cell-like confinement.

Line 258: Panel g conflates “spots” with “proteins”. How was the observation of localized fluorescence reduced to number of proteins?

Line 300: How reasonable is this approximation?

Line 342: The experiments described in this paragraph bring to mind many questions and concerns. I understand the use of lambda DNA as a facile, commonly-used model polymer for fluorescence imaging of confined molecules. This background is not offered to the reader, and so its use comes across as a nonsequitur.

Line 352: Perhaps there are differences significant to transcription, but these are not resolved by the current experimental procedure.

Line 355: What is meant here by “drag force”? Surely, it is rather some combination of fluid flow around the DNA and interactions between the DNA and electric field directly that result in this stretching.

Line 365: What is meant by “classical polymer physics”? Still the sentence is perhaps one of the more lucid statements in the paper.

Lines 362 to 363: It is unclear how the claim of a system out of equilibrium” is relevant to the stated central purpose of the paper to describe the “swelling” of the chromosome during transcription. What is “the system” here?

Or, is the forced claim of a lack of equilibrium an effort to justify the designation of the chromosomes as active gels? The experimental evidence for meaningful measurements of transcriptional dynamics on the chromosomes is weak (e.g.

Supplementary Figure 9) and seems central to the claim of chromosomes as active gels.

Lines 269 to 371: This would be a lovely concluding sentence, if it were true. However, the claim that the paper describes “fundamental effects” is too far a reach. Perhaps “phenomenological effects” would be more accurate.

Line 712: What are “standard electric wires”?

Line 713: How are the microscopy images analyzed specifically? Stating the software package is insufficient to reproduce the analysis. Is the background analyzed? How are the images segmented? Etc.

Supplementary Figure 1 panels c and d: How were the raw micrographs altered in the preparation of the figure, if at all?

Supplementary Figures 2, 3, 4, 5, 6, 7, 8, and 9: How were the raw micrographs altered in the preparation of the figure, if at all?

Supplementary Figure 4 panel c: What is the uncertainty on the lysis curve data?

Supplementary Figure 6 Line 75: Are the data form both experiments shown? How good was the reproducibility?

Supplementary Figure 9: How is a “blob” defined? How is a blob tracked across images over time, such that a (2-dimensional projection of a) “jump distance” can be measured? Define “jump distance’ in the main text for the reader.

Reviewer #6

(Remarks to the Author)
The manuscript describes how the E. coli chromosome swells when transcription is occurring and how MukBEF causes local compaction of the chromosome. The work elegantly combines different approaches to support these findings. Below I have outlined 3 major points and several minor recommendations.

Major points:
1) The authors should show that the 500 nM rifampicin significantly reduces transcription in their system.
2) It was unclear to me how the authors conclude that addition of the lysate compacts the chromosome. From Figure 1, I only see that transcription swells the chromosome, but not that the lysate compacts it. If I understood correctly, both the top and bottom panel in b is in the presence of lysate, the only difference is the transcriptional activity of the lysate. The authors should include the data showing this.
3) It would be interesting to characterize to what extent proteinase K removes all proteins. The RNA polymerase experiment is not sufficient, as I would be more interested in NAPs or BCPs. Conversely, if proteinase K does not remove all proteins, what is the difference between the “proteinated” and “deproteinated” chromosome.

Minor points:
1) The SYBR Green intensity appears lower for proteinated chromosomes (Figure 1F versus Figure 1b), could the authors comment on this?
2) The authors claim that 10% of the ribosomes are chromosome bound, however it is unclear if this is calculated by colocalization and therefore excludes the ribosomes in proximity to the DNA structures. Wouldn’t it make more sense to also include the ribosomes that are in proximity to DNA structures (though not colocalized) as chromosome bound ribosomes.
3) The authors state that that there is very little turnover of bound MukB (line 227-228). The authors should specify how “turnover” is measured.
4) Figure 5 shows that protein expression is similar (even a bit higher) in their system compared to plasmids. How does this align with the finding that only 10% of the ribosomes are chromosome bound?

Version 3:

Reviewer comments:

Reviewer #3

(Remarks to the Author)
This is an excellent manuscript that describes how isolated chromosomes could be manipulated within a flow cell to deduce their size and fine structure. The findings clearly advance the field. The revised manuscript is substantially improved. The authors constructively addressed my previous criticism. I recommend this manuscript for publication.

Reviewer #4

(Remarks to the Author)
The manuscript has been significantly improved and is now suitable for publication.

Reviewer #5

(Remarks to the Author)
The authors have addressed my previous concerns through thoughtful, major revision of their manuscript. The explication and technical rigor are both greatly improved.

To make a minor clarification, consider using a symbol other than the null sign in figures to refer to the chamber diameter, as the null sign is used elsewhere to indicate schematically a negative result.

Reviewer #6

(Remarks to the Author)
The authors have adequately addressed my comments.

Open Access This Peer Review File is licensed under a Creative Commons Attribution 4.0 International License, which permits use, sharing, adaptation, distribution and reproduction in any medium or format, as long as you give appropriate credit to the original author(s) and the source, provide a link to the Creative Commons license, and indicate if changes were made.
In cases where reviewers are anonymous, credit should be given to 'Anonymous Referee' and the source.
The images or other third party material in this Peer Review File are included in the article’s Creative Commons license, unless indicated otherwise in a credit line to the material. If material is not included in the article’s Creative Commons license and your intended use is not permitted by statutory regulation or exceeds the permitted use, you will need to obtain permission directly from the copyright holder.
To view a copy of this license, visit https://creativecommons.org/licenses/by/4.0/

We thank all reviewers for their critical and helpful evaluation. We now significantly revised the manuscript (main text and supplementary information) for logic and flow and performed several further experiments to address the comments, including three main areas:

1) **Compaction of chromosomes in dilute buffer and cell-free gene expression systems**: We now estimate areas of the chromosome transplanted into large, diamond-shaped compartments in a dilute buffer and the cell-free gene expression system, including a comprehensive titration of synthetic crowder PEG. The detailed Python image-processing software is provided as Data Source.
2) **Rifampicin as inhibitor for transcription initiation**: We added bulk experiments to titrate the rifampicin concentration and have also placed a previous publication from our group (Greiss *et al.*, Nat Comm, 2024) at the corresponding position in the main text, reporting on the use of rifampicin to inhibit coupled transcription and translation at the single-DNA level.
3) **Unspecific protein degradation with Proteinase K**: We studied two more examples for different DNA-bound proteins, i.e., MukB-HT and HUα-GFP, and found an identical fast degradation compared to the RNAP (β'-HT) decay when the cell lysis buffer was supplemented with Proteinase K.

Please see our detailed responses (black font with specified sections from the manuscript framed in black boxes) to the specific reviewer comments (blue font) below. The changes in the manuscript are highlighted in red.

Following the significant revision, we have adapted the title of our manuscript: "**Bacterial chromosome conformation and cell-free gene expression in synthetic 2D compartments**".

Reviewer #3 (Remarks to the Author):

This is a very interesting manuscript that describes a procedure for bacterial cell lysis in the confines of a flow cells followed by characterization of released chromosomes in response to transcription/translation and expression of MukB. The authors report that the chromosomes swell during transcription, condense in the presence of MukB, and that ribosomes localize away from the chromosomes. These results agree with previous conclusions that were obtained in live cells or on reconstituted much smaller systems. The main advantage of this system is that the studies are performed in situ on isolated chromosomes. This is a significant advance in the field that has many potential applications.

The weaknesses of the paper include poor organization, lack of clarity in the description of experiments, and insufficient statistical analysis. It is often unclear what was the purpose of experiments, what were the main conclusions and how they relate to the main thread of the paper. Experiments are often poorly described to the point that it is sometimes utterly unclear what has been done. For comparative studies, p-values must be calculated. The number of observed chromosomes is usually unreported. In some cases that are mentioned below, the analysis appeared questionable.

We thank this reviewer for the positive comments. We took the reviewer's critical comments into account, rearranged, revised, and added parts to the manuscript, and improved the organization and clarity. We also added statistical tests where comparisons were done. Please see further details below.

Specific comments.

1. Fig. 1 presents E. coli chromosomes isolated in flow cells. The size distribution of the chromosomes must be shown. Please compare the size or volume of the chromosomes with expectations based on at least some DNA models. A discussion of what limits the size of transplanted chromosomes would be appropriate. Please also compare sizes of chromosomes that were deproteinized or not.

We changed the analysis accordingly and now show the area distribution of chromosomes transplanted into large, diamond compartments in dilute buffer (PBS, as a reference) and the cell-free transcription-translation (TxTl) system, following the treatment with Proteinase K to remove chromosome-bound proteins (see Fig. 5). We also changed the analysis for protein-bound chromosomes without Proteinase K treatment and estimated the area of chromosomes (see Fig. 4d). We modified the corresponding sections in the manuscript.

2. The protocol for depoteinization of the chromosomes involves a treatment with Proteinase K. This protocol does not ensure complete removal of proteins and peptides from DNA. This needs to be reflected in the analysis.

We appreciate the reviewer's insights. We performed several experiments that indicate the effective removal of various types of DNA-binding proteins, including RNAP, MukB, and HUα. The updated section is:

For this, we added the unspecific protease (Proteinase K, PKA) to the lysis buffer. We confirmed protein degradation on the chromosome through the rapid fluorescent signal loss for RNAP-HT (**Fig. 5a, Supplementary Fig. 10, and Movie S4** without PKA against **Movie S5** with PKA), HUα-GFP, and MukB-HT (**Fig. 5a, Supplementary Fig. 11, Movie S6**). All three examples demonstrated that the deproteination of the chromosomes was effective for different types of DNA-binding proteins.

3. Fig. 1b and 1f compare compartments with active (top half) and inhibited (bottom half) transcription. The comparison would be more instructive if the top and bottom parts of the figures showed the same compartment.

We thank the reviewer for pointing out this issue. The corresponding figure (now Fig. 5) was significantly changed during the revision process and now includes new data around a PEG titration. The figure update has also clarified this comment.

4. Line 738- indicate the extent of transcription inhibition by 500 nM rifampicin at the conditions of experiment. Please include a reference or show data.

We placed the corresponding reference of a previous publication from our group (Greiss *et al.*, Nat Comm, 2024) and also included an experiment with rifampicin (Supplementary Fig. 4), inhibiting the GFP production in bulk cell-free TxTl expression experiments at 500 nM. The updated section in the main text:

In addition, we repeated the experiment using a full TxTl system supplemented with 500 nM rifampicin, an inhibitor of transcription initiation, thereby blocking activity of endogenous RNAPs from the TxTl system (**Supplementary Fig. 4**) *(48)* and preventing any competition with native RNAPs.

5. Line 99 “we could observe chromosomes self-organizing into an intricate arrangement, likely due to steric hindrance by the neighboring chromosomes”. The shown data are insufficient to infer that the size of the chromosomes is dictated by the steric hindrance. A similar experiment at a reduced cell density would help evaluate the effect of the steric hindrance.

We agree that the current data do not sufficiently support the conclusion about steric hindrance. We therefore removed the speculative statement from the manuscript to avoid overinterpretation. We thank the reviewer for helping us refine the interpretation.

6. For all figures showing averages, how many chromosomes were quantified? Are the observed differences statistically significant?

We now extensively specify the number of chromosomes in the figure captions and compare the area distributions with statistical methods, stating the p-value in the figures. Please see the corresponding sections in the updated manuscript.

7. Please provide details on quantification of SG-I intensity and its normalization. The question is especially important because chromosomes are often merged.

We appreciate this observation. During the revision process, we changed the quantification and now estimate the area of chromosomes using a custom image processing pipeline described in the Methods section and provided as Python Notebook. We removed the SG-I intensity analysis from the manuscript.

8. Fig. 1. SG-I intensity is a proxy for DNA accessibility and amount. It is not a good proxy for chromosome size. Please show the data for chromosome size.

We again agree with the reviewer and now perform an area estimate with a custom image processing pipeline as described in the Methods section and provided as Python Notebook. We report on chromosome areas throughout the manuscript. We updated the Methods section:

> The cell-free chromosomes were segmented with the SYBR Green I using StarDist v0.9.1 *(66)* for estimating the chromosome areas. We used the pre-trained StarDist model "2d_versatile_fluo" for 2D to segment chromosomes in the large diamond-shaped compartments. We manually selected subregions inside the large compartments to exclude the fluorescence signal of non-lysed cells from the analysis. The detailed algorithm is provided as Python Notebook in the Data Source.

9. Fig. S4. Please indicate which proteins were tagged in each experiment.

We updated this Supplementary Figure (now Supplementary Fig. 2) and clarified which proteins were tagged in each experiment by indicating and outlining the figure layout with labels and a gray box for each bacterial strain.

10. Fig. 3c Please mark the time periods when the electric field was applied.

We updated the panel in this figure (now Fig. 2c).

11. Line 196. "Using the RNAP-labeled E. coli cells, the chromosome entered the capillary with the RNAP signal covering it continuously, apparently unaffected by the different DNA densities (Fig. 3e, Supplementary Fig. 5b)". The statement sounds ambiguous, please rephrase. According to Fig. 3e, RNAP density is proportional to the HU density, not unaffected by it.

We thank the reviewer for pointing out this ambiguity. We clarified this section in the main text:

> The HT-labeled RNAP and HT-labeled ribosomes had markedly different signatures from MukB-HT (**Fig. 2e,f, and Supplementary Fig. 3b**). The RNAP covered the DNA along its entire contour, with a signal roughly linearly proportional to the DNA signal, both at the regions of blobs and elsewhere.

12. Fig. S6 needs diagrams of experiments or clearer explanations. Fig S6a- what happens between 1 min and 30 min? What is shown in Fig. S6c? How many chromosomes were analyzed and how did RNAP-HT make it into the graph given that it was not labeled for the experiment in Fig. S6ab.

We updated and clarified the Supplementary Figure and split it into two separate figures (now Supplementary Figs. 7 and 8). We now state the number of experiments conducted to reproduce the results. We repositioned Supplementary Fig. 6c into Fig. 4 and explicitly describe the experiment in the main text:

> We observed a slow exponential decay of the MukB-HT signal with a time constant of ~7 min (versus ~3 min for RNAP in separate experiments), with ~30% remaining bound to the chromosome (~7% for RNAP) after 25 minutes (**Fig. 4b, Movie S3**).

And we describe the statistics in the caption of Fig. 4b:

> **b)** The fluorescence signals of MukB-HT and RNAP (β'-HT) in separate experiments, each averaged over 4 chromosomes from two independent biological experiments in large compartments. The dashed colored lines show fits to mono-exponential decays ($t_{MukB\text{-}HT}$=6.89 ± 0.20 min and t_{RNAP}=2.63 ± 0.03 min) with an immobile fraction ($A_{MukB\text{-}HT}$ =0.27 ± 0.01 and A_{RNAP}=0.071 ± 0.002).

13. Movie S4. What is shown on the three panels of the movie? This is a common problem with all movies, they need more extensive legends.

We agree and thank the reviewer for their comment. The movie (now Movie S3) shows a double-color fluorescence time lapse with native MukB-HT clusters (in magenta, rightmost panel) bound to chromosomes from donor cells in large compartments, transitioning from cell lysis buffer to the cell-free TxTl system. The TxTl system is supplemented with the DNA intercalator SYBR Green-I (SG-I) to label the chromosomes (in cyan, middle panel). The MukB-HT channel and SG-I channel are overlaid as a double-color movie in the leftmost panel.

We updated and clarified all movie descriptions. The description for Movie S3:

> Supplementary Movie 3: Double-color time-lapse of transplanted chromosomes (after cell lysis) and native MukB-HT during transition from cell lysis buffer to a cell-free expression reaction containing 200 nM SYBR Green I. Chromosomes (SYBR signal, cyan) and MukB-HT (fluorescent HaloTag signal, magenta) were derived from MG1655 mukB-HT donor cells. The movie shows a small region in a large steady-state compartment. The time stamp and scale bar are shown in the movie.

14. Line 235. Please describe the model in Fig 4d in a separate paragraph. This model integrates data described in multiple experiments presented in Fig 2-4.

Given the reviewer's concern and the scope of the manuscript, we updated and removed the model from Fig. 4 to focus on our main findings around transplanted chromosomes in cell-free gene expression systems.

15. How many MukB clusters did the authors observe in a chromosome?

We manually identified MukB clusters emerging from donor cells directly after cell lysis. We have added the 5 examples to Supplementary Figure 7b and show one lysis example in Movie S2. We describe the data in the main text:

> After lysis of cells containing fluorescently labeled MukB-HT, we identified between 2-9 (median of 5, N=5) chromosome-bound MukB clusters on single chromosomes (**Movie S2**, **Supplementary Fig. 7b**).

16. Line 271, "Instead, we used the fluorescently labeled RNAP and ribosomes bound to the proteinated chromosome from the source bacteria". This sentence is unclear, please rephrase. How was the experiment depicted in Fig 5 done?

We thank this reviewer for pointing out the ambiguity. We updated the corresponding text section and described the experiment in this figure (now Fig. 3):

> The stable and uniform binding of native RNAPs on the chromosome in buffer suggested that upon lysis, the RNAPs were caught in arrest of transcriptional elongation, most likely on the ~700 σ_{70}-regulated housekeeping genes of the genome *(46)*. We asked whether the bound RNAPs maintained their functionality and could resume activity, and if so, use them to estimate the rate of mRNA transcription in the TxTl system. We introduced the TxTl system into the microfluidic chip, tracking the fluorescence signal of chromosome-bound native RNAPs.

17. The data in Fig 5a and 5b are very similar to those in Fig. 3e, 3f and arrive at the same conclusion. Please revise this section to avoid redundancy.

We removed these panels from the figure (now Fig. 3).

18. Line 276, "We introduced the cell-free gene expression system, tracking the expression dynamics through RNAP." This sentence is too cryptic. Please explicitly state what was done. Was it a cell extract with RNAP or something else? Also, this sentence begins a description of a new experiment, put it into a new paragraph.

We thank this reviewer for pointing out the ambiguity. We updated the corresponding text section and described the experiment in this figure (now Fig. 3):

> The stable and uniform binding of native RNAPs on the chromosome in buffer suggested that upon lysis, the RNAPs were caught in arrest of transcriptional elongation, most likely on the ~700 s_{70}-regulated housekeeping genes of the genome *(46)*. We asked whether the bound RNAPs maintained their functionality and could resume activity, and if so, use them to estimate the rate of mRNA transcription in the TxTl system. We introduced the TxTl system into the microfluidic chip, tracking the fluorescence signal of chromosome-bound native RNAPs.

19. Line 283 "To gain further support, we omitted chemical energy from the reaction ..." What does this mean in practice?

We updated the corresponding section in the main text:

> To verify transcriptional activity, we used a TxTl system lacking the energy buffer containing nucleotides for transcription (**Methods**) and observed a slower release of native RNAPs from the chromosome, as expected (**Fig. 3c**).

20. The paragraph on lines 302-314 attempts to calculate the free energy generated by ongoing transcription in the isolated chromosomes. Please complete the argument by providing the calculated free energy. Please also keep in mind that RNAP can use up to 44% of the energy of nucleotide hydrolysis for mechanical work, but the actual number is often less than that.

We thank this reviewer for their insights. During the extensive revision of the manuscript, we removed the simplified calculation and focused on our reported experiments.

21. The purpose of experiments in the section "Gene expression dynamics on a cell-free chromosome" is unclear. Please state the objective in the beginning of the section and conclusions in the end.

We updated the section title to "**Measuring protein synthesis from a single chromosome**" and the corresponding sections in the main text to clarify our objective. At the beginning of the section, we state:

> In the next set of experiments, we introduced an *E. coli* endogenous cell-free TxTl system with the aim of measuring the rate of protein synthesis from a single *E. coli* chromosome in the semi-open compartment.

At the end of the section, we state:

> This result contrasts with the amplification reported in bulk TxTl with rates of ~10^3 protein per hour and DNA template *(48)* but agrees with recent work on coupled TxTl from a single DNA molecule in a highly dilute limit *(47)*.

22. Supplemental figure 9c shows time lapse micrographs of diffusing "semi-conserved structures" in the chromosome. However, the conservation of these structures is limited to

much shorter time intervals than the duration of the experiment. The placement of the asterisk in Fig. S9c seems arbitrary to this reviewer. This raises concerns about veracity of the entire experiment on diffusion rates.

We thank this reviewer for their insights and agree with their concerns. We removed this section from the revised manuscript to focus on our main experiments.

23. Fig S9a outlines an experiment with MukB. However, this experiment is never mentioned in the main text of the article.

We updated this figure (now Fig. 9) and describe the experiment in the main text:

> As a control, we verified the low bulk MukB concentrations in the TxTl system reported in previous studies *(52)*. This was done by preparing two TxTl systems, one prepared from the *E. coli* strain MG1655 mukB-HT and another one from an unmodified strain, measuring MukB-HT fluorescence signals only slightly above background fluorescence signals (**Supplementary Fig. 9**).

24. Neither objectives nor conclusions of section “DNA displacement fluctuations under cell-free gene expression” (lines 329-358) were clear to this reviewer.

We agree with this reviewer and decided to remove this section from the revised manuscript, focusing on our main experiments.

25. Discussion restates the general knowledge in the field. Instead, it should highlight the main findings of the study and summarize how these findings enhance the state of the art.

We thank the reviewer for this suggestion. We revised the Discussion section and described our achievement and findings: 1) synthetic 2D compartments to reconsitute chromosome dynamics outside a cell 2) chromosome conformation in buffer and cell-free TxTl systems, with varying concentrations of the synthetic crowder PEG, and 3) the protein synthesis rate in cell-free TxTl systems at the single-DNA level and its implications. Please see the revised section in the manuscript.

Reviewer #4 (Remarks to the Author):

In this manuscript, Greiss and colleagues demonstrate that in vitro transcription can induce the swelling of E. coli chromosomes confined in diamond-shaped semi-open compartments when native binding proteins are degraded. They further show that chromosome-bound proteins can "protect" the chromosomes from debunching, leading to the formation of blob-like structures. The authors map the spatial distribution of condensin proteins, RNA polymerases, and ribosomes within the system and analyze gene expression dynamics on a cell-free chromosome. The concept of transcription-dependent chromosome swelling is new and interesting. Overall, I think the manuscript shows some important aspects in sufficient depth and is very worthwhile for considering publication in Nature Communications.

We thank this reviewer for their positive evaluation.

Concerns:

1. The authors describe chromosomes as active gels organized by molecular machines, but this claim appears speculative, as no direct experimental evidence is provided to support it. Similarly, the influence of macromolecular crowding on chromosome dynamics is not adequately addressed. While the authors state, “But without losing the expression activity, we could not directly test the response of DNA swelling by transcription to different crowding conditions,” it is worth noting that adding certain amounts of PEG (a crowding agent) to cell-free systems can enhance gene expression. The role of macromolecular crowding in chromosome dynamics should be further explored.

We thank this reviewer for their insights and agree with the speculative nature around active gels. We therefore decided to remove “active gels” from the manuscript and revised the manuscript for flow and clarity. We also added various experiments that show cell-free chromosome areas in dilute buffer and cell-free TxTl system at various PEG concentrations, with and without rifampicin to inhibit transcription initiation. Please see updated Fig. 5 and the corresponding section “**Chromosome conformation affected by molecular crowding and transcription**”.

2. In Fig 1b, direct evidence of in vitro transcription occurring from the chromosomes is lacking. Without this, the functional relevance of rifampicin in the experiments remains unclear.

We used rifampicin in a previous paper of our group (Greiss *et al.*, Nat Comm, 2024) to inhibit transcription in the cell-free TxTl system. We apologize for the missing reference for this

statement, which we added in the revised manuscript. To further support our claim, we performed an extra bulk expression experiment to again verify complete transcription inhibition by rifampicin at 500 nM (Supplementary Fig. 4). We clarified the corresponding section in the main manuscript:

> In addition, we repeated the experiment using a full TxTl system supplemented with 500 nM rifampicin, an inhibitor of transcription initiation, thereby blocking activity of endogenous RNAPs from the TxTl system (**Supplementary Fig. 4**) *(48)* and preventing any competition with native RNAPs.

3. In Fig 3a, are HUα-labeled chromosomes in liquid or gel states? Why do they recover from the stretched state so quickly? The rapid recovery of chromosomes from the stretched state raises questions about the driving forces behind this process. Further clarification is needed.

A detailed mechanical analysis of the chromosome stretching and relaxation dynamics was not performed because of the potential electrosmotic flows induced by the electric field inside the thin capillary. Further quantitative work needs to be done to understand all physical aspects and derive a general mechanical model. However, we note that similar relaxation times were reported in theoretical modeling (see Ref. by Ruben et al., *Biophysical Journal*, 2023 in revised manuscript). We updated the section:

> The HUα-labeled chromosome stretched and relaxed reversibly in about a second, consistent with relaxation times reported on mitotic DNA *(39)*, supporting the notion that the blobs were stable structures and that the chromosome was not fragmented on larger scales (**Fig. 2c**, **Supplementary Fig. 3a**).

We also discuss potential electroosmotic flow in the capillary in the Methods section:

> A further analysis of chromosome stretching and relaxation dynamics was not performed because of potential electroosmotic flows induced by the electric field inside the thin capillary. Future quantitative work needs to consider all physical aspects to derive a general mechanical chromosome model.

4. The molecular mechanisms underlying transcription-induced chromosome swelling remain poorly understood, which is the weakest part of the manuscript. Elucidating these mechanisms would greatly strengthen the study.

Considering all reviewer comments, we decided to rearrange the manuscript for better flow and presentation: To reconstitute chromosome dynamics in a cell-free gene expression

system in semi-open compartments, and insights into the chromosome conformations. We agree that a molecular mechanism would strengthen the manuscript. However, we believe this is beyond the current scope of this manuscript.

Reviewer #5 (Remarks to the Author):

“Transcription-dependent swelling of a bacterial chromosome” presents an interesting and timely study at integrating some of arguably the most compelling intellectual and technological currents at the cutting edge of biotechnology broadly and synthetic biology specifically. The overall motivation for this work is irreproachable. The field requires guidance—at this stage, empirical or phenomenological will do—to understand and control the engineering implications of design choices around biomolecular interactions for confined genomes and cell-free expression systems.

We thank the reviewer for their enthusiastic comments on our research direction.

However, the paper would benefit from major revision to clarify the main purpose and argument throughout the manuscript. The stated conclusions and speculated impact of the work are generally overreaching and should be reconsidered to more closely match the actual results of the study. The paper should be revised to clarify the argument and selection of the experimental studies in support of that argument. A main challenge is accomplishing this task lies in the highly multidisciplinary nature of the study, which puts tremendous responsibility on the authors to be thoughtful in their word choices and clear in their explication. Phrases like “single molecule” and “active gels” are used, without clear explanation or their relevance to the main argument of the work. With some care and attention, this paper can make a substantive contribution to the field. I recommend major revision prior to accepting for publication.

We agree with this reviewer and significantly revised the manuscript to clarify our research motivation and results. Please see the detailed responses below.

- What are the noteworthy results?

The observation of chromosome behavior confined to chip-based synthetic cells using cell-free expression systems is a welcome addition to the literature.

- Will the work be of significance to the field and related fields? How does it compare to the established literature? If the work is not original, please provide relevant references.

Yes.

- Does the work support the conclusions and claims, or is additional evidence needed?

- Are there any flaws in the data analysis, interpretation and conclusions?

See detailed comments below.

- Do these prohibit publication or require revision?

No, and yes, respectively. See detailed comments below.

- Is the methodology sound? Does the work meet the expected standards in your field?

Yes, the expectations of the field are generally met, except with regard to descriptions of the measurements and equipment calibration. The image analysis, in particular, requires more detail to follow the progression from photons detected at a sensor to segmented images to reduced data quantities with associated measurement uncertainties.

- Is there enough detail provided in the methods for the work to be reproduced?

Yes, given that the cited references and resources are also consulted.

Detailed comments by manuscript line:

Line 20: “Autonomous biomaterials” seems to be a curious singular application area to highlight here. Why this example only, as opposed to others? What does “autonomous biomaterials” mean exactly here? Consider using clearer language.

We agree with this reviewer and removed this phrase from the revised manuscript.

Lines 22 to 23: Reaching the “single molecule limit” is a bold claim. Which single molecule? Imaging to resolve a single chromosome is decidedly more tractable than, say, a single protein. Or, is this meant to refer to a single fluor? Surely, this cannot be the case, given that the main claims of the paper pertain to chromosomal organization and conformation. The reader, however, cannot yet know this, and so I recommend clarifying this.

We imaged single HT proteins produced from plasmids and chromosomes, now shown in Fig. 4e. We, however, agree with this reviewer that the main focus of the work was not about single molecules, but chromosome dynamics and conformations. We therefore removed this statement in the introduction, better highlighting our focus on bacterial chromosome conformation and cell-free gene expression in compartments.

Lines 26 to 27: This sentence is confusing, because it seems to be attempting—with limited success—to convey too many disparate ideas. Consider breaking this into two separate sentences. The logic here is implicit and needs to be made explicit. This requires care and matters, because these ideas represent, I understand, the heart of the results presented in this paper.

We carefully revised the abstract in the current manuscript, hopefully better explaining our work and results.

Line 30: What is meant here by “nonequilibrium” exactly? In what way is the swelling “nonequilibrium”? The overall effect of the swelling can be amenable to an equilibrium description, even if the underlying molecular mechanisms that result in the emergent behavior of the swelling are the result of dynamic processes.

We apologize for the unclear phrasing. During the extensive revision of the manuscript, we remove this wording. Please see the revised manuscript for details.

Line 32: The claim of “detailed balance locally broken” requires clarification. Even the “arrow” of time itself is not so at certain length and time scales. Similarly, detailed balance is a convenience applicable to its own certain length and time scales. It is unclear to which results this statement refers and how this statement relates to the main claims of the paper of transcription-dependent swelling.

We carefully revised the abstract in the current manuscript, better explaining our work and results around *E. coli* chromosome dynamics and conformation in cell-free TxTl systems. Please see the revised manuscript for details.

Upon further reflection, perhaps a discussion on relevant time and length scales is missing. The paper seems to be aggressively forcing the conclusion of chromosomes as "active gels" for an undisclosed reason. This is all fine and good, but consider stating why this designation is edifying: does the fundamental formalism around active gels lead to new insight for synthetic biology or enable previously impossible engineering capability? The paper does not say.

We agree with this reviewer and revised the manuscript, removing the phrase "active gels". Instead, we focus on the method development and our main findings around cell-free gene expression from bacterial chromosomes and their *in vitro* conformation.

Line 35: Consider removing "the" and replacing with "a". Certainly, this is _a_ frontier but only one of many. Refrain from overstating the importance of this work. And, what is "bottom-up synthetic biology"? This term is not well-defined by the community. Consider revising for clarity. Is this meant to refer to so-called "bottom up" approaches to creating synthetic cells?

We certainly agree with this reviewer. We replaced "the" with "a". Otherwise, we decided to keep "bottom-up synthetic biology" in the manuscript, considered as the broader research area beyond synthetic cells:

Programming artificial cells with single chromosomes is a frontier for bottom-up synthetic biology.

Line 42: The word choice of "transactions" reads strangely in English. Is this the best word here?

Following established literature on DNA-related processes (by Kornberg, von Hippel, etc.), we decided on "DNA transactions" as a general concept for protein-DNA interactions, including but not limited to gene expression.

Line 44: This statement assumes that artificial cells will not confine chromosomes to a small volume. This need not be the case, and other researchers, as the authors are well aware, do indeed labor to create artificial cells of the same physical dimensions as extant, naturally-evolved cells.

We agree with this comment and changed the section to:

Embedding *E. coli* chromosomes in semi-open synthetic compartments could advance our understanding by releasing the chromosome from its confined volume and/or complex, intertwined dynamics *in vivo*.

Line 46: Consider clarifying for the reader what is meant here exactly by "active". Physically active? Biologically active? Both? Something else entirely? It is unclear.

We agree and clarified the section:

Further engineering, with the required knowledge of such fundamental processes, could establish chromosomes as biologically active genomes for future autonomous artificial cells *(16)*.

Line 49: Consider adding "biologically" before "active". See comment for Line 46.

We changed the section:

Further engineering, with the required knowledge of such fundamental processes, could establish chromosomes as biologically active genomes for future autonomous artificial cells *(16)*.

Line 53 to 54: This is such an important consideration. How was DNA breakage assayed and observed in this study? Or, what justifies its neglect?

We appreciate this comment. Firstly, the stretching experiments of chromosomes using an electric field suggested that major fragmentation could be prevented by gently lysing bacterial cells on a chip. The corresponding section:

To test for major fragmentation of the chromosome and stability of the genome organization in the lysis buffer, we applied a weak alternating electric field across the compartment to pull and release chromosome regions into and from the thin capillary (**Fig. 2a,b, and Movie S1**). The HUα-labeled chromosome stretched and relaxed reversibly in about a second,

consistent with relaxation times reported on mitotic DNA *(39)*, supporting the notion that the blobs were stable structures and that the chromosome was not fragmented on larger scales (**Fig. 2c**, **Supplementary Fig. 3a**).

Secondly, we have now performed control experiments (see Fig. 5d) that show long-term, reversible compaction-decompaction cycles in the cell-free TxTl system upon increasing and decreasing PEG concentrations. The reversibility suggested that chromosomes remained intact despite potential residual nuclease activity in the TxTl systems. The corresponding section:

The long-term reversibility further suggested that chromosomes maintained their integrity throughout the experiments, which might have been compromised by photo-induced DNA damage and residual nuclease activity in the TxTl system.

Line 63: “High resolution” is meaningless in this sentence. Quantitatively, what resolution, or, better yet, resolution sufficient to resolve...what exactly?

We agree and have removed this phrase from the introduction.

Line 65: Check the verb tense.

We significantly revised this paragraph in the introduction and replaced this sentence.

Line 66: Is “energy source” the most correct and rigorous description for this? Perhaps “effective energy”?

We significantly revised this paragraph in the introduction and replaced this sentence.

Is it the transcription itself that is swelling the chromosome? Or, is the swelling the combined effect of many factors contributing to complex chromosomal dynamics, to wit: DNA melting and changes in conformation, entropy, and other associated polymer dynamics of the chromosome; electrostatic consequences of bound proteins that affect the solvent quality and associated polymer attributes related to conformation; effect of the physical confinement?

We significantly revised the manuscript, putting the focus on our achievements around cell-free gene expression from chromosomes and their conformation. We agree that elucidating

the molecular mechanism behind transcription-dependent swelling is intriguing, but beyond the scope of the current manuscript. We added a section to the Discussion:

> The dose-response function of chromosome conformation with the synthetic crowder PEG is intriguing. The fact that a compaction transition in buffer occurs at an increased PEG concentration than in the TxTl system shows that ~25 mg ml^{-1} background proteins in the TxTl system suffice to compact the chromosome *(52)*. Adding rifampicin to inhibit transcription initiation pushed the compaction transition to lower PEG levels. Furthermore, the conformation of the chromosome was slightly expanded without rifampicin. These results suggest that transcription acting globally on the chromosome effectively swells the DNA, in possible agreement with recent simulations *(55–57)*. The general experimental approach presented here could be developed further to address this intriguing observation.

"Transcription" may be a catch-all phrase used by biologists for a biomolecular process, but using it to describe the physical phenomenon of counterbalanced forces acting to result in polymer behavior seems inappropriate and even, perhaps, misleading. Rather, consider breaking down for the reader what underlying biomolecular dynamics and biophysics are meant here by "transcription". Do not deliberately obfuscate matters for the reader.

We agree with this reviewer that transcription itself involves complex biophysical processes. Elucidating which effect contributes to the chromosome swelling could be addressed in the future. Please also see the comment above for the updated section in the Discussion.

Line 77: What is the dye molecule to DNA base pair ratio?

In order to reduce effects from SG-I on the chromosome conformation, we reduced the SG-I concentration to minimal levels (0.1-0.2x of the recommended concentration) while still being able to detect cell-free chromosomes. We followed the recommendations by Kolbeck *et al.*, NAR (2021) as referenced in this manuscript. They found the dissociation constant K_D ~ 0.3 μM between dsDNA and SG-I, estimating a ~30% occupancy of chromosomes by SG-I (1:3 dye and DNA ratio). We updated the corresponding section in the Methods part:

> The lysis buffer was exchanged under a flow of 0.5 μL min^{-1} by a cell-free gene expression system supplemented with 100-200 nM SYBR Green I (0.1-0.2x supplier recommended working concentration, Thermo Fisher Scientific, to get a 1:3 dye molecule to DNA ratio considering a dissociation constant K_D ~ 0.3 μM) *(64)* for experiments in the large compartments, and, in the case of inhibited transcription, 500 nM rifampicin (15 μM stock solution was stored in DMSO at -20 °C).

Page 3 Figure 1 panels b, d, f, and h: How were the raw micrographs altered in the preparation of the figure, if at all?

The figure outline was changed during the revision of the manuscript (now Fig. 5). Except for Fig. 1d (right panel) showing a deconvoluted image of chromosomes, no images were altered in any way throughout the manuscript. We added a brief description in the Methods section:

The raw fluorescence images shown in the figures were processed by adjusting the minimum and maximum values to the same levels when comparing images in similar conditions using Fiji v1.0 software, and we mapped these 16-bit images into 8-bit for visualization on computer screens.

We state the convolution in Fig. 1d in the figure caption:

The fluorescence image was deconvolved with Huygens v24.04 to highlight the blob-like structures.

Line 100: What is the difference between the chromosomes distancing from each other due to "steric hinderance" versus, say, entropically-driven separation? Technically, "steric hinderance" refers to the effect of a molecule's physical structure negatively impacting its ability to react chemically. Is this what is meant in this case? I doubt it. Consider using a more appropriate term.

We agree with this comment and the ambiguity around "steric hindrance". We removed this statement in the revised manuscript.

Lines 106 to 107: Was the response of the microscope to increased fluorescence within a given area of the detector characterized for linearity within the range of intensities relevant to the studies presented?

We worked in the linear regime of the EM-CCD camera (by adjusting exposure times, EM gain, and the excitation power, please see details in the Methods section). Nonetheless, we removed the SG-I intensity measurement and now estimate the chromosome area.

Line 111: The use of "surprising" to begin this paragraph seems disingenuous. This seems unsurprising, especially given the reference. Was the compaction due to "transcription

inhibition"? Or, was the compaction due to changes in the solvent quality for the chromosomal DNA due to the addition of rifampicin and its associated effects on molecules in the solvent? Is this behavior a transient that is then followed, as in cyto, by an expansion of the chromosome?

We agree and have removed this statement from the revised manuscript.

Line 130: This is perhaps an opportune moment in this review to inquire into how photobleaching, photonicking, and other photodamage to the DNA was mitigated and measured. I see that the devices are incubated in a nitrogen environment, the reason for which I do not believe has been given. Was this to reduce photodamage during imaging, assuming that the nitrogen passed through the PDMS of the device?

We agree, and we carefully reduced excitation power and SG-I concentrations to minimize photo-induced damage. We now also show reversibility of chromosome compaction and swelling by increasing and decreasing PEG concentrations throughout the course of a >1-h long experiment with a constant excitation (Fig. 5d), indicating that the overall integrity of chromosomes is maintained for a long time. We added a section to the main text:

> The long-term reversibility further suggested that chromosomes maintained their integrity throughout the experiments, which might have been compromised by photo-induced DNA damage and residual nuclease activity in the TxTl system.

The nitrogen-flushed box is used to maintain a constant humidity (the nitrogen was directed through a custom-built water bath) and prevent drying out of the microfluidic chip. We further explain this detail in the corresponding Methods section:

> We constructed a custom stage chamber and flushed it with humid N_2 to maintain a constant humidity and prevent the microfluidic chip from drying out.

Furthermore, what is the surface chemistry of the devices? How much non-specific binding was observed, and how did it impact (or not) the fluorescence measurements or movement of the chromosome? What are the approximate Debye lengths on the surfaces, and how might that have impacted (or not) the diffusive exchange of components of the cell-free system on which some of the experiments rely?

We described in the "Design, fabrication, and preparation of microfluidic chips" methods section the passivation of the microfluidic chip with a Silane-PEG monolayer (developed in our group and verified extensively). The passivation with Silane-PEG is a standard in single-

molecule and bulk biophysical studies to prevent biomolecule adsorption to surfaces. Still, in some cases, minor, nonspecific adsorption was observed.

The height of a PEG monolayer can be estimated with its molecular weight of 3.4 kDa (see the reference in manuscript by Buxboim *et al.*, Small, 2007) and a monomer weight of 62 Da (Chemical formular of monomer: $C_2H_6O_1$), with a size of ~0.3 nm (e.g., doi:10.1088/1367-2630/1/1/006). About 55 monomers are in one PEG chain (=3400/62), amounting to a PEG contour length of ~17nm (=55*0.3nm). The contour length is ~90-fold (=1500/17) smaller than the microfluidic compartment height used in this study. Debye lengths in salty buffer (such as phosphate-buffered saline buffer, PBS) were reported to be shorter than 10 nm, within the range of the PEG monolayer. Because of these short PEG and Debye lengths, we could neglect their effects on chromosome conformation and material diffusion inside the compartments.

Line 141: Is the deconvolution method given in the Methods? I see insufficient detail given in the Methods section.

The convolution was done with Huygens v24.04 and is now stated in the caption of Fig. 1:

> The fluorescence image was deconvolved with Huygens v24.04 to highlight the blob-like structures.

Figure 3: Please indicate how the raw micrographs were processed to create the images included in the figure.

We added a brief description of image processing to the Methods section:

> The raw fluorescence images shown in the figures were processed by adjusting the minimum and maximum values to the same levels when comparing images in similar conditions using Fiji v1.0 software, and we mapped these 16-bit images into 8-bit for visualization on computer screens.

The processing of the kymograph in Fig. 2b is described in the Methods section:

> The fluorescence profile along the capillary was measured using Fiji v1.0 and plotted as a kymograph using the command "Reslice". The kymograph was loaded in Python v3.7 and processed using scikit-image v0.17.2 and NumPy v1.20.3 (see processing software provided as Python Notebook in the Data Source).

Line 183: This paper is an interesting and opportune amalgam of several disciplines, and not all readers are likely to be conversant in all of them. Consider stating what MukBEF is and why it was chosen, to help motivate its alleged significant contribution to determining the chromosome conformation.

We added a description of MukB in the main text:

(i) MukB-HT – the bacterial condensin complex MukBEF, proposed as active loop extruder and key genome organizer that localizes across the chromosome as discrete clusters *(41–45)*, each consisting of ~36 MukB proteins *(46)* that entrap DNA locally.

Line 186: 20% of what? Please specify.

We revised this section in the current manuscript and updated it:

The low occupancy of MukB on the chromosome is consistent with *in vivo* experiments reporting a DNA-bound MukB fraction of ~20% with a short residence time of ~50 seconds for MukB on DNA *(46)*.

Line 238: I strongly recommend labeling this proposed model as “empirical” or “phenomenological”, because the model is not derived from first principles or even explicitly grounded in existing and relevant understanding in related fields, notably polymer physics. While the utility of this proposed model is unclear to me, others may find it useful in the future for qualitative guidance on chromosome behavior under cell-like confinement.

We agree with the reviewer and have significantly revised this part of the manuscript. We removed the simplified model.

Line 258: Panel g conflates “spots” with “proteins”. How was the observation of localized fluorescence reduced to number of proteins?

We thank the reviewer for pointing out the confusion. We changed the caption of Fig. 3:

e) Exemplary dual-color fluorescence image of a chromosome labeled with HUα-GFP, and two surface-captured HA-tagged HaloTag proteins labeled through fluorogenic MaP655-Halo dye (white arrows). The dashed white line outlines the compartment and capillaries connected with two main flow channels. **f)** Time traces of arrival time and counts of HA-HaloTag protein spots captured and recorded on the surface during the TxTl reaction from a

chromosome in the compartment. **g)** The rate of HaloTag proteins expressed in a TxTl reaction with single plasmids, chromosomes, and without DNA.

Line 300: How reasonable is this approximation?

We changed the section during the revision of the manuscript, and now state:

Interestingly, the same result was obtained using single shorter recombinant DNA molecules (plasmids) with the same cassette (Methods, **Fig. 3e-g, Supplementary Figs. 5 and 6**). This result contrasts with the amplification reported in bulk TxTl with rates of ~10^3 protein per hour and DNA template *(49)* but agrees with recent work on coupled TxTl from a single DNA molecule in a highly dilute limit *(48)*.

Line 342: The experiments described in this paragraph bring to mind many questions and concerns. I understand the use of lambda DNA as a facile, commonly-used model polymer for fluorescence imaging of confined molecules. This background is not offered to the reader, and so its use comes across as a nonsequitur.

We thank the reviewer for describing their concerns. We agree and removed the experiments to focus on the main findings of our work about cell-free chromosomes.

Line 352: Perhaps there are differences significant to transcription, but these are not resolved by the current experimental procedure.

We agree and removed the experiments to focus on the main findings of our work.

Line 355: What is meant here by "drag force"? Surely, it is rather some combination of fluid flow around the DNA and interactions between the DNA and electric field directly that result in this stretching.

We removed this section during the significant revision of our manuscript. We hope the reviewer appreciates our additional work on cell-free chromosomes in buffer and the cell-free TxTl systems, adjusting macromolecular crowding levels with the synthetic crowder PEG.

Line 365: What is meant by "classical polymer physics"? Still the sentence is perhaps one of the more lucid statements in the paper.

We removed the statement during the extensive revisions of our manuscript.

Lines 362 to 363: It is unclear how the claim of a system out of equilibrium" is relevant to the stated central purpose of the paper to describe the "swelling" of the chromosome during transcription. What is "the system" here?

We removed the statement during the revisions of our manuscript.

Or, is the forced claim of a lack of equilibrium an effort to justify the designation of the chromosomes as active gels? The experimental evidence for meaningful measurements of transcriptional dynamics on the chromosomes is weak (e.g. Supplementary Figure 9) and seems central to the claim of chromosomes as active gels.

We agree and removed the experiments to focus on the main findings of our work. We significantly revised the manuscript, putting the focus on our achievements around cell-free gene expression from chromosomes and their conformation.

Lines 269 to 371: This would be a lovely concluding sentence, if it were true. However, the claim that the paper describes "fundamental effects" is too far a reach. Perhaps "phenomenological effects" would be more accurate.

We revised this section and removed the claim.

Line 712: What are "standard electric wires"?

We thank the reviewer for pointing out the missing information. These were standard copper wires. The updated section:

Electrodes were constructed from standard electric copper wires and installed into a second pair of microfluidic in- and outlet ports.

Line 713: How are the microscopy images analyzed specifically? Stating the software package is insufficient to reproduce the analysis. Is the background analyzed? How are the images segmented? Etc.

We included the Python processing software with exemplary data as an interactive notebook for the chromosome (labeled by HUα-GFP) pulling experiment using an electric field and the chromosome segmentation (labeled by SYBR Green I) in large compartments. We modified the Methods section. In the section "***Local pulling of transplanted chromosomes with an electric field***":

> The kymograph was loaded in Python v3.7 and processed using scikit-image v0.17.2 and NumPy v1.20.3 (see processing software provided as Python Notebook in the Data Source).

And in the section of "***Image segmentation of chromosomes in large compartments***":

> The cell-free chromosomes were segmented with the SYBR Green I using StarDist v0.9.1 *(66)* for estimating the chromosome areas. We used the pre-trained StarDist model "2d_versatile_fluo" for 2D to segment chromosomes in the large diamond-shaped compartments. We manually selected subregions inside the large compartments to exclude the fluorescence signal of non-lysed cells from the analysis. The detailed algorithm is provided as Python Notebook in the Data Source.

Supplementary Figure 1 panels c and d: How were the raw micrographs altered in the preparation of the figure, if at all?

Except for the convolution of the fluorescence image shown in Fig. 1d (right panel), no image alteration was done. We describe the general approach in the Methods section:

> The raw fluorescence images shown in the figures were processed by adjusting the minimum and maximum values to the same levels when comparing images in similar conditions using Fiji v1.0 software, and we mapped these 16-bit images into 8-bit for visualization on computer screens.

Supplementary Figures 2, 3, 4, 5, 6, 7, 8, and 9: How were the raw micrographs altered in the preparation of the figure, if at all?

Please see our response above.

Supplementary Figure 4 panel c: What is the uncertainty on the lysis curve data?

We performed multiple cell lysis experiments and plotted individual lysis curves in the figure, helping to estimate the differences between individual cell lysis events. The updated section in Supplementary Fig. 2c:

**c)** The cell lysis curves were produced from single bacteria trapped in large compartments (N cells, as indicated in the bar graph and shown as black circles), with the red bar as median. We tracked the fluorescence signal and computed the ratios before and after cell lysis to find the fraction of chromosome-bound proteins. At least two biologically independent experiments were performed to produce the data.

Supplementary Figure 6 Line 75: Are the data form both experiments shown? How good was the reproducibility?

We revised the figure and now show the data in Fig. 4b. We performed the experiment twice in biologically independent experiments and averaged over a total of 4 chromosomes with good reproducibility (the underlying data is provides as Source Data file):

**b)** The fluorescence signals of MukB-HT and RNAP (β'-HT) in separate experiments, each averaged over 4 chromosomes from two independent biological experiments in large compartments.

Supplementary Figure 9: How is a "blob" defined? How is a blob tracked across images over time, such that a (2-dimensional projection of a) "jump distance" can be measured? Define "jump distance' in the main text for the reader.

We agree with the reviewer about the ambiguity of a "blob" in the previous Supplementary Fig. 9. We removed this figure during the revision of the manuscript, and instead added a comprehensive set of experiments focusing on the chromosome conformation in different conditions, i.e., dilute buffer and cell-free TxTl system, and different PEG concentrations.

Reviewer #6 (Remarks to the Author):

The manuscript describes how the E. coli chromosome swells when transcription is occurring and how MukBEF causes local compaction of the chromosome. The work elegantly combines different approaches to support these findings. Below I have outlined 3 major points and several minor recommendations.

We thank the reviewer for their positive evaluation.

Major points:

1) The authors should show that the 500 nM rifampicin significantly reduces transcription in their system.

We added the rifampicin experiment showing complete inhibition of protein synthesis at 500 nM (now Supplementary Fig. 4a). We also placed a reference to our previous work (Greiss *et al.*, Nat Comm, 2024) on coupled transcription-translation at the single-DNA level, reporting on rifampicin's inhibitory effects in the cell-free TxTl system. We updated section:

In addition, we repeated the experiment using a full TxTl system supplemented with 500 nM rifampicin, an inhibitor of transcription initiation, thereby blocking activity of endogenous RNAPs from the TxTl system (**Supplementary Fig. 4**) *(48)* and preventing any competition with native RNAPs

2) It was unclear to me how the authors conclude that addition of the lysate compacts the chromosome. From Figure 1, I only see that transcription swells the chromosome, but not that the lysate compacts it. If I understood correctly, both the top and bottom panel in b is in the presence of lysate, the only difference is the transcriptional activity of the lysate. The authors should include the data showing this.

We thank the reviewer and agree with the comment. We have added additional experiments that compare chromosome conformations in dilute buffer and the cell-free TxTl conditions, highlighting that the background proteins present in the TxTl system compact chromosomes. The data of chromosome areas in dilute buffer and cell-free TxTl, each with an extensive PEG titration, are now shown in Fig. 5. The section in the main text is updated:

The response with increasing PEG in dilute buffer revealed that chromosomes maintained an area of ~20 μm^2 at low PEG concentrations, which decreased with PEG and showed a sharp transition to the most compact conformation (~3 μm^2) at 9% PEG without a further compaction at 10% PEG (**Fig. 5c-e, buffer**). We then measured the compaction level of the chromosomes with a TxTl system having a protein background concentration of ~25 mg ml^{-1} *(53)* and 2% PEG for optimal TxTl activity (**Supplementary Fig. 4b)** *(54)*.

And further clarified in the Discussion:

The dose-response function of chromosome conformation with the synthetic crowder PEG is intriguing. The fact that a compaction transition in buffer occurs at an increased PEG concentration than in the TxTl system shows that ~25 mg ml^{-1} background proteins in the TxTl system suffice to compact the chromosome *(52)*.

3) It would be interesting to characterize to what extent proteinase K removes all proteins. The RNA polymerase experiment is not sufficient, as I would be more interested in NAPs or BCPs. Conversely, if proteinase K does not remove all proteins, what is the difference between the "proteinated" and "deproteinated" chromosome.

We performed the Proteinase K treatment on chromosomes with for two other DNA-binding proteins, i.e., MukB-HT and HUα-GFP, and could observe the identical (to RNA polymerase) fast drop in fluorescence signal. We modified the section in the manuscript and attached Movies S4 and S5 for visualization:

In the final set of experiments, we studied chromosome conformations without any native proteins transferred from the donor cell. For this, we added the unspecific protease (Proteinase K, PKA) to the lysis buffer. We confirmed protein degradation on the chromosome through the rapid fluorescent signal loss for RNAP-HT (**Fig. 5a, Supplementary Fig. 10, and Movie S4** without PKA against **Movie S5** with PKA), HUα-GFP, and MukB-HT (**Supplementary Fig. 11, Movie S6**). All three examples demonstrated that the deproteination of the chromosomes was effective for different types of DNA-binding proteins.

Minor points:

1) The SYBR Green intensity appears lower for proteinated chromosomes (Figure 1F versus Figure 1b), could the authors comment on this?

We thank the reviewer for the comment. We changed the figures during the revision of the manuscript. The regions of interest from the large compartments are now shown in Fig. 5. Instead of fluorescence intensity, we now analyze the images by segmentation to estimate chromosome areas and provide the Python processing software in the Data Source.

2) The authors claim that 10% of the ribosomes are chromosome bound, however it is unclear if this is calculated by colocalization and therefore excludes the ribosomes in proximity to the DNA structures. Wouldn't it make more sense to also include the ribosomes

that are in proximity to DNA structures (though not colocalized) as chromosome bound ribosomes.

We thank the reviewer for pointing out the ambiguity. We measured a median of ~10% ribosomes remained localized to the chromosomes after cell lysis in the lysis buffer. The updated section is:

> By measuring the HT signal before and after cell lysis with these strains, we could estimate the occupancy of the MukB, RNAP, and ribosomes on the chromosome to be about 15%, 65% and 10%, respectively (**Fig. 2d-f**).

3) The authors state that that there is very little turnover of bound MukB (line 227-228). The authors should specify how "turnover" is measured.

We thank the reviewer for pointing out the unclear phrasing and clarified the section in the manuscript:

> We observed a slow exponential decay of the MukB-HT signal with a time constant of ~7 min (versus ~3 min for RNAP), with ~30% remaining bound to the chromosome (~7% for RNAP) after 25 minutes (**Fig. 4b, Movie S3**).

4) Figure 5 shows that protein expression is similar (even a bit higher) in their system compared to plasmids. How does this align with the finding that only 10% of the ribosomes are chromosome bound?

We apologize for the confusion in this part. We significantly revised the manuscript to clarify the workflow. The 10% ribosomes account for the chromosome-bound ribosomes after cell lysis from the donor cell in the cell lysis buffer. We did not measure the ribosome dynamics in the cell-free TxTl system because of the very high bulk ribosome concentration and low chromosome occupancy.

We also provide extensive statistical tests (Fig. 3g) for comparing the protein synthesis rates and show that protein synthesis rates between plasmids and chromosomes are not statistically significant ($p>0.05$). The revised section in the manuscript:

> Interestingly, the same result was obtained using single shorter recombinant DNA molecules (plasmids) with the same cassette (Methods, **Fig. 3e-g, Supplementary Figs. 5 and 6**).

We again thank all reviewers for their positive evaluation and for helping us improve the manuscript. Please see our detailed response (black font) to the reviewer #5 comment (blue font) below.

Reviewer #3 (Remarks to the Author):

This is an excellent manuscript that describes how isolated chromosomes could be manipulated within a flow cell to deduce their size and fine structure. The findings clearly advance the field. The revised manuscript is substantially improved. The authors constructively addressed my previous criticism. I recommend this manuscript for publication.

Reviewer #4 (Remarks to the Author):

The manuscript has been significantly improved and is now suitable for publication.

Reviewer #5 (Remarks to the Author):

The authors have addressed my previous concerns through thoughtful, major revision of their manuscript. The explication and technical rigor are both greatly improved.

To make a minor clarification, consider using a symbol other than the null sign in figures to refer to the chamber diameter, as the null sign is used elsewhere to indicate schematically a negative result.

We thank the reviewer for pointing out the ambiguity. We changed the null sign in Figs. S5 and S6 to “DIA” as a standard abbreviation for diameter in technical drawings.

Reviewer #6 (Remarks to the Author):

The authors have adequately addressed my comments.
